# Supplementary material for: Evaluating the feasibility and acceptability of virtual group exercise for older adults delivered by trained volunteers: the ImPACt study protocol
Source: BMJ Open. 2022 Feb 1;12(2):e052631. doi: 10.1136/bmjopen-2021-052631 (PMC8808406; doi:10.1136/bmjopen-2021-052631)
Supplement: Supplementary data [file bmjopen-2021-052631supp001.pdf]

BMJ Publishing Group Limited (BMJ) disclaims all liability and responsibility arising from any reliance placed on this supplemental material which has been supplied by the author(s)

ImPACt Volunteer Competencies

These competencies have been developed to enhance club members care during online exercise sessions. They have been broken down into separate competencies to ensure you feel equipped to work with older adults to promote their activity, whilst maintaining the individuals and your own safety. Each box should be dated and signed by yourself and the supporting therapist or researcher.

Name: **Date started:** **Date completed:**

Skill / Knowledge	Criteria	Training given	Skill practiced with supervision on peers	Skill practiced with supervision on patient	Skill completed (signed by therapist / researcher)
Personal safety awareness	Wear appropriate clothing and footwear to be comfortable when exercising.				
	Ensure your exercise space is set up safely, including a sturdy chair, and the space is free of obstacles.				
	Ensure you are well enough to exercise and know when exercise should be avoided.				
Basic patient safety	Demonstrate what to do if a participant feels unwell. Stop exercise. Participants should not exercise if they have a fever or any new or worsening symptoms.				
	If participants have symptoms indicative of an emergency situation e.g., chest pain, dizziness, or shortness of breath that does not go away upon cessation of exercise, then call 999.				
	CALL FOR HELP IF YOU FEEL IT IS AN EMERGENCY<br>If there is an emergency situation alert a staff member, who will support you.				

Lim SER, *et al. BMJ Open* 2022; 12:e052631. doi: 10.1136/bmjopen-2021-052631

	Staff members will have access to participants emergency contact info.				
	Demonstrate what to do if a participant experiences pain during exercise. Stop and review the participants position and technique. Offer an alternative exercise if pain persists, or suggest they miss out the exercise and rest. Advise they seek advice from their GP if pain persists.				
Pre exercise checks	Demonstrate proficiency with online set up and use of an online platform (e.g., Zoom).				
	Introduction to participants / group. Explain role and overview of exercise session.				
	Determine participants readiness and safety to exercise. How are you? How are you feeling?				
	Ensure participants have a safe exercise set-up, including a sturdy chair, water nearby for hydration, and the room is clear of obstacles.				
	Ensure patient has appropriate clothing and footwear to exercise.				
Prescribed exercises	To demonstrate an understanding of the main components of an exercise session, including warm up, conditioning, and cool down.				

	To prompt and demonstrate correct technique for lower limb chair exercises.				
	To prompt and demonstrate correct technique for upper limb chair exercises.				
	To demonstrate verbal coaching proficiency, including clear feedback on exercise technique and rationale to participants.				
	To demonstrate ability to encourage and motivate participants to exercise.				
	To demonstrate understanding of exercise progressions.				
Documentation	Proficiency completing participant registers.				
	Proficiency completing adverse events log.				
