# Supplementary material for: Evaluating the feasibility and acceptability of virtual group exercise for older adults delivered by trained volunteers: the ImPACt study protocol
Source: BMJ Open. 2022 Feb 1;12(2):e052631. doi: 10.1136/bmjopen-2021-052631 (PMC8808406; doi:10.1136/bmjopen-2021-052631)
Supplement: Author's manuscript [file bmjopen-2021-052631.draft_revisions.pdf]

BMJ Open is committed to open peer review. As part of this commitment we make the peer review history of every article we publish publicly available.

When an article is published we post the peer reviewers' comments and the authors' responses online. We also post the versions of the paper that were used during peer review. These are the versions that the peer review comments apply to.

The versions of the paper that follow are the versions that were submitted during the peer review process. They are not the versions of record or the final published versions. They should not be cited or distributed as the published version of this manuscript.

BMJ Open is an open access journal and the full, final, typeset and author-corrected version of record of the manuscript is available on our site with no access controls, subscription charges or pay-per-view fees (http://bmjopen.bmj.com).

If you have any questions on BMJ Open's open peer review process please email info.bmjopen@bmj.com

BMJ Open

Evaluating the feasibility and acceptability of virtual group exercise for older adults delivered by trained volunteer: the ImPACt study protocol

Journal:	*BMJ Open*
Manuscript ID	bmjopen-2021-052631
Article Type:	Protocol
Date Submitted by the Author:	21-Apr-2021

Complete List of Authors:	Lim, Stephen; University of Southampton, Academic Geriatric Medicine; University of Southampton, NIHR ARC Wessex<br>Meredith, Samantha; University of Southampton, Academic Geriatric Medicine; University of Southampton, NIHR ARC Wessex<br>Agnew, Samantha; The Brendoncare Foundation<br>Clift, Esther; Southern Health NHS Foundation Trust<br>Ibrahim, Kinda; University of Southampton, Academic Geriatric Medicine, Faculty of Medicine; University of Southampton, NIHR ARC Wessex<br>Roberts, Helen; University of Southampton, Academic Geriatric Medicine; University of Southampton, NIHR ARC Wessex

Keywords:	GERIATRIC MEDICINE, QUALITATIVE RESEARCH, REHABILITATION MEDICINE

SCHOLARONE™
Manuscripts

Title: Evaluating the feasibility and acceptability of virtual group exercise for older adults delivered by trained volunteer: the ImPACt study protocol

Protocol version 1.0

Corresponding author: Dr Stephen Eu Ruen Lim

Email: s.e.lim@soton.ac.uk

Address: Mailpoint 807, Academic Geriatric Medicine, University of Southampton,

Southampton General Hospital, Tremona Road,

Southampton, United Kingdom, SO16 6YD

Tel: 02381206131

Co-authors:

Dr Samantha Jane Meredith; University of Southampton, Southampton, UK

Samantha Agnew; The Brendoncare Foundation, Winchester, UK

Esther Clift; Southern Health NHS Foundation Trust, Southampton, UK

Dr Kinda Ibrahim; University of Southampton, Southampton, UK

Professor Helen Roberts; University of Southampton, Southampton, UK

Key words:

Exercise, Older adults, volunteer, physical activity

Word count: 2896 words

For peer review only - http://bmjopen.bmj.com/site/about/guidelines.xhtml

Abstract

Introduction

Physical activity is important for healthy ageing. Despite strong evidence on the benefits of physical activity for health and well-being, physical inactivity remains a significant problem among older adults. There is little evidence on the use of trained volunteers to lead online exercise groups. The aim of this study is to determine the feasibility and acceptability of implementing an online volunteer-led group exercise for older adults.

Methods

A quasi-experimental mixed methods approach will be used in this feasibility study. A training programme will be developed to train the volunteers to safely deliver group exercises online to older adults aged >65 years(n=30). The primary outcome of this study is the feasibility of implementing the online volunteer-led group exercise. This will be assessed by determining the number of volunteers recruited, trained, and retained at the end of the study, the number of physical activity sessions delivered and completed by participants, and the fidelity of the exercise intervention delivered by volunteers. Secondary outcome measures include physical activity levels, physical function, and quality of life, measured at baseline and at 6 months. Qualitative interviews will be conducted with volunteers(n=5), exercise group participants(n=10), and family members(n=5) to explore their views of the online group exercise.

Analysis

Simple descriptive statistics will be used to describe participant characteristics, the feasibility of the study and the impact of the intervention on health outcomes. Qualitative data will be analysed using an inductive thematic analysis approach. A toolkit will be developed to support future implementation of the intervention.

Ethics and Dissemination

This study has received ethical approval from the university's ethics and research governance committee. Findings from this study will be published in peer-reviewed journals and will be made available to service users and voluntary organisations who may be interested in implementing the intervention.

Trial registration number

For peer review only

For peer review only - http://bmjopen.bmj.com/site/about/guidelines.xhtml

Clinicaltrials.gov: NCT04672200

For peer review only

For peer review only - http://bmjopen.bmj.com/site/about/guidelines.xhtml

Article summary

Strengths and limitations of this study

- This is a novel study exploring the use of trained volunteers to deliver online exercise groups for older adults.
- It addresses the issue of physical inactivity among older people, a problem which has been exacerbated by the COVID-19 pandemic.
- A mixed methods approach will support the development of a toolkit for future implementation.
- For safety reasons, volunteers will deliver seated exercises in online groups.
- The exclusion of older adults who are not able to provide consent limits the generalisability of this study.

For peer review only

Introduction

Physical activity is important for healthy ageing. Some of the benefits of regular physical activity for older people include reduction in falls(1), better physical functioning(2), and improvement in well-being(3). The recommended physical activity level for older adults aged 65 years and above is to achieve 150 minutes of moderate physical activity in a week, with 2 days of strength training(4). This is often expressed as 30 minutes of brisk walking or equivalent activity five days a week, although 75 minutes of vigorous intensity activity spread across the week, or a combination of moderate and vigorous activity are also suggested(5).

Despite the proven benefit of physical activity, physical inactivity remains a significant problem among older adults. Studies in the UK have shown that less than 7% of community-dwelling older adults achieve the recommended weekly physical activity level(6, 7). The COVID-19 pandemic has exacerbated the problem of physical inactivity. Rules on social distancing and shielding of vulnerable groups have resulted in increased physical inactivity among older people, with potentially widespread deconditioning.

The rise of the use of technology during the pandemic may provide a solution to address the problem of physical inactivity among older people. Older adults are embracing modern technology more than ever before and are increasingly familiar with the use of devices and online platforms to stay in touch with their family members and friends(8, 9). This presents an opportunity to develop online physical activity interventions for older adults to promote increased physical activity. A review of reviews conducted by McGarrigle et al found low to moderate evidence that physical activity interventions delivered through the internet and related technologies may be effective in increasing physical activity in older adults in the short term(10). Online group exercises have been shown to be feasible for older adults(11). However, there is little evidence on the use of trained volunteers to lead such exercise groups.

Volunteers play an integral part within many health and social care organisations. They enrich patient experience, help deliver high quality services, and support communities in living healthier lives. A report by the King's Fund on volunteering has highlighted that the support volunteers provide can be of particular value to those who rely most heavily on services, such as people with multiple long-term conditions or mental health problems(12). There is increasing evidence to suggest that with proper training, volunteers can take on more direct roles in supporting older adults particularly with nutrition(13) and mobility(14).

For peer review only - http://bmjopen.bmj.com/site/about/guidelines.xhtml

Aims

This study aims to determine the feasibility and acceptability of training volunteers to deliver online group exercises to community-dwelling older adults. The specific objectives of this study are to:

1. Develop a training programme for volunteers to lead online exercise classes
2. Determine the feasibility of recruiting and training volunteers to deliver the online intervention
3. Determine the acceptability of the online intervention to volunteers, older adults, and their carers
4. Explore barriers and facilitators to the implementation of the online intervention

Methods and Analysis

Study design

A quasi-experimental mixed methods approach will be used to determine the feasibility and acceptability of implementing online volunteer-led exercise sessions in community clubs. This feasibility study will be conducted at community-clubs focussed on promoting social interaction among older people. Feasibility studies are used to determine whether an intervention is appropriate for further evaluation, to determine sample sizes for controlled trials and to assess whether the ideas and findings can be shaped to be relevant and sustainable. In this study, the feasibility of training volunteers to promote increased physical activity among community-dwelling older adults will be assessed. The acceptability of the intervention will also be examined through qualitative interviews to explore the views and experiences of volunteers, older adults participating in the study, and their carers or family members. The impact of the intervention on physical activity levels and functional outcomes will also be measured. This will help inform the sample size calculation for future effectiveness trial.

Participant recruitment

Volunteer recruitment and training

The research team will be invited by club staff to attend the virtual clubs to introduce the study. Club volunteers and members who are interested in leading the exercise groups will indicate their interest to staff members or approach the research team directly. The participant information sheet

For peer review only - http://bmjopen.bmj.com/site/about/guidelines.xhtml

and the consent form will be sent through the post. Club members are invited to contact the research team for further discussions about the study. Once the research team have received the signed consent form, volunteers will be invited to participate in the online training. To deliver online exercises to 30 exercise participants, it is estimated that at least 12 volunteers will be needed to deliver 6 online group exercise classes. Volunteers will be paired up to deliver the group exercises. There are no inclusion or exclusion criteria for the volunteers.

The volunteer training programme will be led by a member of the research team with a background in sport and exercise science. The training programme will be developed based on clinical expertise from therapists and current evidence including experience from a recently completed hospital-based study(14). The training session will include training on personal safety and safety of participants, and the exercise and mobility protocols. Each volunteer will receive two group training sessions and further one to one sessions will be offered to volunteers who wishes to have further coaching. Competency assessments based on a checklist will be conducted by the research team to ensure that volunteers are competent in delivering the exercises online. Throughout the study period, fidelity checks will be conducted to ensure that the volunteers are delivering high quality exercise sessions. Volunteers will also receive training on how to document adverse events that occur during the exercise classes.

Regular meetings for the volunteers will be held throughout the study period to provide them with ongoing support from the research team and the organisation running the clubs. The group meetings will provide an avenue for volunteers to discuss and share their experiences, ask questions, and provide suggestions to improve the intervention.

Exercise participant recruitment

Club members who are interested in participating in the online group exercise will indicate their interests to club staff or approach the research team directly. Participant information sheet and consent forms will be posted to club members.

The inclusion criteria for the group exercise participants are: adults aged 65 years and older, able to walk with or without a walking aid, and able to provide written consent (Table 1). Participants who are actively participating in other online exercise classes will be excluded. The aim is to recruit 30 participants to receive the online exercise intervention.

For peer review only - http://bmjopen.bmj.com/site/about/guidelines.xhtml

Intervention

The intervention consists of a once weekly volunteer-led online group home-based exercise, for a duration of 30 minutes. The exercises are mainly seated exercises and will focus on strengthening upper and lower limbs and enhancing whole body range of motion and flexibility, tailored specifically to meet the needs of older adults and to minimise the risks of injury. Participants will be encouraged by the volunteers to progress by increasing repetitions and to gently improve range of motion. Besides exercising, participants are also encouraged to increase mobility levels. The exercise sessions will be piloted in one club before being extended to other clubs.

Outcomes measures

Volunteer characteristics such as age, gender, employment status, previous volunteering experience, and experience in delivering group exercises will recorded. Participant characteristics including age, gender, domicile status, body mass index, co-morbidities and number of medications will be recorded.

Primary outcomes

The primary outcome of this study is the feasibility of implementing volunteer-led exercise sessions in the community for older adults. This will be assessed by determining:

1. the number of volunteers recruited, trained, and retained at the end of the study,
2. the number of physical activity sessions delivered and completed by participants, and
3. the fidelity of the exercise intervention delivered by volunteers.

Secondary outcomes

The secondary outcome measures include measurement of physical activity levels and performance of activities of daily living (ADL). Physical activity levels will be measured using the Community Health Model Activities Program for Seniors (CHAMPS) questionnaire(15). Performance of activities of daily living will be measured using the Barthel Index(16). EuroQOL will be used to measure quality of life(17), and the SARC-F questionnaire will be used as a measure for sarcopenia(18). PRISMA-7 will be used to characterise participants' frailty status. The 26-point Telephone Mini-mental state examination (TMMSE) will be used to assess participants' cognitive function. Data collection will be conducted remotely through telephone to comply with social distancing measures.

For peer review only - http://bmjopen.bmj.com/site/about/guidelines.xhtml

These outcome measures will be recorded at recruitment using remote assessment formats (e.g., telephone-based) and repeated at 6 months. Cost analysis will be conducted to determine the cost of implementing this intervention.

Safety and adverse events

To determine the safety of the intervention, adverse events that occur as a result of the intervention will be recorded. Any injuries or symptoms developed directly as a result of the exercises will be recorded as an adverse event. Volunteers will be trained to document any adverse events and report it back to the research team.

Acceptability of the intervention

Interviews will be conducted among older adults, their family members or caresr, volunteers, and those involved in recruiting participants and training volunteers, to determine the acceptability of the training and the intervention and to explore barriers and enablers to the implementation of the intervention. Interviews will be semi-structured to help guide conversation whilst allowing participants the flexibility to elaborate and reflect on any meaningful experiences(19).

Older adults will be selected by purposive sampling to share their thoughts and views regarding the implementation of the volunteer-led physical activity session(20). To ensure a wide range of views are included in the interviews, participants will be selected to include male and female participants, different clubs, and a representative age range. The aim is to interview at least two participants per club, with a total number of 10 participants. Family members and carers (n=5) of the exercise participants will also be invited to participate in the interviews. Their views and experience will provide valuable insight into the acceptability of the intervention and the potential barriers and enablers for a future wider implementation. Interviews will be conducted via telephone or online (e.g., Zoom, Skype) depending upon participant preference.

Semi-structured focus groups will also be conducted with volunteers leading the intervention. Discussions among volunteers are likely to generate a wider perspective of the implementation process and may provide insight into the group narrative on their experiences in delivering the intervention and their interaction with the research participants. The aim is to interview at least one volunteer from each club, with a total number of five volunteers. Volunteers will be interviewed

For peer review only - http://bmjopen.bmj.com/site/about/guidelines.xhtml

individually via telephone or as a group online (e.g., Zoom, Skype) depending upon participant preference.

The semi-structured interviews will consist of several key open-ended questions that help define the areas explored but allow the interviewer or interviewee to expand and diverge with the aim of pursuing or developing an idea with more depth. The interview schedules will be underpinned by Normalisation Process Theory (NPT)(21). The interviews will seek to explore the views of older adults and volunteers on the exercise sessions, the barriers and facilitators to the intervention and suggestions for future implementation studies. The interviews will be audio-recorded for data collection purposes.

The NPT provides a set of sociological tools to understand and explain the social processes through which new or modified practice of thinking, enacting, and organizing work are operationalized in healthcare and other institutional settings(21). The use of an implementation theory is useful as it helps researchers identify, describe and explain important elements of the implementation process(22). NPT was chosen as the framework that underpinned this study as it was systematic in its approach, easy to understand and provided a proven framework to evaluate the implementation process of this study. The online toolkit available for this implementation theory also made the application of NPT convenient and practical(23). The interview schedules were developed based on the four components of NPT:

I. Coherence: meaning and sense-making.
II. Cognitive participation: this explores the likelihood of all participants being committed and engaged with the intervention.
III. Collective action: the consideration of the work that participants do to make the intervention function.
IV. Reflexive monitoring: which involves participants appraising or reflecting on the intervention.

Data analysis plan

Quantitative data analysis

Data collected will be double entered into a secured database for analysis. Statistical analysis will be conducted using the statistical software SPSS Statistics 25. Descriptive statistics -median (IQR); mean (SD); number (%) – will be used to analyse the numbers of volunteers recruited, trained and retained, as well as the type and extent of progression of the seated exercise, and duration of their

For peer review only - http://bmjopen.bmj.com/site/about/guidelines.xhtml

activity, and patients' adherence to the intervention. Analysis of the above outcome measures will be used to develop an assessment of the feasibility of delivering this intervention. Adherence to the intervention and reasons for non-adherence will be reported.

To determine suitability for a future, fully-powered effectiveness study of the intervention, outcome measures recorded at baseline will be compared to measurement at 6 months to determine if PA intervention had an impact on physical activity levels measured by CHAMPS, functional outcomes including performance in activities of daily living (Barthel Index), and quality of life (EuroQol). The distribution of each outcome measure will be assessed for normality and described using parametric or non-parametric statistics accordingly. A basic cost-analysis of the training programme will be carried out, costing the time of clinical staff involved in delivering the training.

Qualitative data analysis

Data collected from the interviews will be transcribed verbatim and analysed with an inductive thematic analysis approach(24). This approach includes: Phase 1 – familiarising with the data, Phase 2 – generating initial codes, Phase 3 – searching for themes, Phase 4 – reviewing themes, Phase 5 – defining and naming themes, and Phase 6 – producing the report. Analysis of qualitative data will be conducted using either Microsoft Word, or with the help of NVIVO, depending on the amount of data collected. A sample of transcribed text will be read and coded separately and then together by two researchers to create a coding manual that applies to the entire set of transcripts. The codes will be analysed to generate concepts and ideas to determine the acceptability of the intervention, and to identify facilitators and barriers to the implementation process. The codes act as tags or labels to help catalogue key concepts embedded within the raw data. From the codes, themes will be developed to reflect the views and experiences of the community-dwelling older adults and volunteers regarding the online physical activity intervention.

Patient and public involvement

PPI involvement was sought in the development of this protocol. Visits were made to two clubs and discussions were held among club volunteers and club attendees about this project. Their views on what type of activities and the frequency of the activities were explored. Club attendees were also asked about what outcomes were important to them, which will be included in the study.

SA who is a co-applicant in this study, will also represent the views of club members and volunteers. She is the head of clubs for the organisation and is experienced in coordinating clubs. She has an overview on how the clubs are conducted and her input on the practical aspects of implementing

For peer review only - http://bmjopen.bmj.com/site/about/guidelines.xhtml

the intervention will be invaluable. Her practical experience gained through interaction with club members and volunteers will help ensure that their views are represented.

Two additional PPI representatives will be invited to be part of the study management group led by the PPI lead for the Ageing and Dementia theme within the Wessex ARC. This group will aim to meet up quarterly to discuss the study progress and will be involved in the design of the research, discussion of the results and dissemination of research findings. PPI has also been involved in the preparation of public-facing materials and study documents.

Conclusion

Findings from this study will provide better evidence on the use of online interventions to promote increased physical activity among older adults. The use of quantitative and qualitative methodologies will help explore the feasibility and acceptability of this intervention and support the development of a toolkit for a cost-effectiveness trial to support future implementation.

(Word count: 2479)

Ethics and dissemination

Ethical and safety considerations

This study has received ethical approval from the University of Southampton ethics and research governance committee (ID:52967.A1). Data will be stored on university repository PURE and will be made available to other researchers on request.

Dissemination plan

Findings from this study will be published in peer-reviewed journals and presented at national and international scientific platforms. A toolkit will be developed to support future implementation plans.

Author Contributions

SL, EC, SA and HCR were involved in the conception and trial design. SM will lead the volunteer training and be involved in data collection, and analysis. All authors contributed in the preparation of this article.

For peer review only

For peer review only - http://bmjopen.bmj.com/site/about/guidelines.xhtml

Funding statement

The National Institute for Health Research (NIHR) Applied Research Collaboration Wessex funded this research. The views expressed are those of the authors and not necessarily those of the NHS, the NIHR or the Department of Health. SL, SM, KI, and HCR received support from the NIHR Applied Research Collaboration (ARC) Wessex and the University of Southampton. HCR received support from the NIHR Southampton Biomedical Research Centre. The University of Southampton NIHR Academic Clinical Lectureship scheme supported SL.

Competing Interests

The authors have no competing interests to declare.

Table 1 Inclusion and exclusion criteria

Inclusion Criteria
Older adults aged > 65 years<br>Able to walk, with or without a walking aid<br>Able to provide written consent
**Exclusion Criteria**
Actively participating in other online exercise classes

For peer review only

References

1. Sherrington C, Fairhall N, Kwok W, Wallbank G, Tiedemann A, Michaleff ZA, et al. Evidence on physical activity and falls prevention for people aged 65+ years: systematic review to inform the WHO guidelines on physical activity and sedentary behaviour. International Journal of Behavioral Nutrition and Physical Activity. 2020;17(1):144.
2. Halaweh H, Willén C, Svantesson U. Association between physical activity and physical functioning in community-dwelling older adults. European Journal of Physiotherapy. 2017;19(1):40-7.
3. Fox KR, Stathi A, McKenna J, Davis MG. Physical activity and mental well-being in older people participating in the Better Ageing Project. European Journal of Applied Physiology. 2007;100(5):591-602.
4. Foster C, Reilly J, Jago R, Murphy M, Skelton D, Cooper A, et al. UK Chief Medical Officers' Physical Activity Guidelines. GOV.UK: UK Department of Health and Social Care 2019 07/09/2019.
5. Sparling PB, Howard BJ, Dunstan DW, Owen N. Recommendations for physical activity in older adults. BMJ. 2015;350(jan20 6):h100-h.
6. Harris TJ, Owen CG, Victor CR, Adams R, Cook DG. What factors are associated with physical activity in older people, assessed objectively by accelerometry? British journal of sports medicine. 2009;43(6):442-50.
7. Jefferis BJ, Sartini C, Lee IM, Choi M, Amuzu A, Gutierrez C, et al. Adherence to physical activity guidelines in older adults, using objectively measured physical activity in a population-based study. BMC public health. 2014;14(1):382-.
8. Marston HR, Genoe R, Freeman S, Kulczycki C, Musselwhite C. Older Adults' Perceptions of ICT: Main Findings from the Technology In Later Life (TILL) Study. Healthcare (Basel). 2019;7(3):86.
9. Barbosa Neves B, Franz R, Judges R, Beermann C, Baecker R. Can Digital Technology Enhance Social Connectedness Among Older Adults? A Feasibility Study. Journal of applied gerontology : the official journal of the Southern Gerontological Society. 2019;38(1):49-72.
10. McGarrigle L, Todd C. Promotion of Physical Activity in Older People Using mHealth and eHealth Technologies: Rapid Review of Reviews. J Med Internet Res. 2020;22(12):e22201.
11. Baez M, Khaghani Far I, Ibarra F, Ferron M, Didino D, Casati F. Effects of online group exercises for older adults on physical, psychological and social wellbeing: a randomized pilot trial. PeerJ. 2017;5:e3150.
12. Naylor C, Mundle C, Weaks L, Buck D. Volunteering in health and care: Securing a sustainable future 2013 . Available from: http://www.kingsfund.org.uk/publications/volunteering-health-and-care. (Last accessed: 20/04/2021)
13. Roberts HC, De Wet S, Porter K, Rood G, Diaper N, Robison J, et al. The feasibility and acceptability of training volunteer mealtime assistants to help older acute hospital inpatients: the Southampton Mealtime Assistance Study. Journal of clinical nursing. 2014;23(21-22):3240-9.
14. Lim S, Ibrahim K, Dodds R, Purkis A, Baxter M, Rogers A, et al. Physical activity in hospitalised older people: the feasibility and acceptability of a volunteer-led mobility intervention in the SoMoVe study. Age Ageing. 2020 27;49(2):283-291.
15. Hekler EB, Buman MP, Haskell WL, Conway TL, Cain KL, Sallis JF, et al. Reliability and validity of CHAMPS self-reported sedentary-to-vigorous intensity physical activity in older adults. J Phys Act Health. 2012;9(2):225-36.
16. Wade DT, Collin C. The Barthel ADL Index: A standard measure of physical disability? International Disability Studies. 1988;10(2):64-7.
17. Rabin R, Charro Fd. EQ-SD: a measure of health status from the EuroQol Group. Annals of Medicine. 2001;33(5):337-43.

For peer review only - http://bmjopen.bmj.com/site/about/guidelines.xhtml

18. Ida S, Kaneko R, Murata K. SARC-F for Screening of Sarcopenia Among Older Adults: A Meta-analysis of Screening Test Accuracy. Journal of the American Medical Directors Association. 2018;19(8):685-9.
19. Gill P, Stewart K, Treasure E, Chadwick B. Methods of data collection in qualitative research: interviews and focus groups. Br Dent J. 2008;204(6):291-5.
20. Palinkas LA, Horwitz SM, Green CA, Wisdom JP, Duan N, Hoagwood K. Purposeful sampling for qualitative data collection and analysis in mixed method implementation research. Administration and policy in mental health. 2015;42(5):533-44.
21. May CR, Mair F, Finch T, MacFarlane A, Dowrick C, Treweek S, et al. Development of a theory of implementation and integration: Normalization Process Theory. Implementation science : IS. 2009;4(1):29-.
22. May C. Towards a general theory of implementation. Implementation Science. 2013;8(1):18-.
23. Normalization Process Theory Toolkit [Available from: http://www.normalizationprocess.org/npt-toolkit/. (Last accessed: 20/04/2021)
24. Braun V, Clarke V. Using thematic analysis in psychology. Qualitative Research in Psychology. 2006;3:77-101.

For peer review only

For peer review only - http://bmjopen.bmj.com/site/about/guidelines.xhtml

Reporting checklist for protocol of a clinical trial.

Based on the SPIRIT guidelines.

Instructions to authors

Complete this checklist by entering the page numbers from your manuscript where readers will find each of the items listed below.

Your article may not currently address all the items on the checklist. Please modify your text to include the missing information. If you are certain that an item does not apply, please write "n/a" and provide a short explanation.

Upload your completed checklist as an extra file when you submit to a journal.

In your methods section, say that you used the SPIRITreporting guidelines, and cite them as:

Chan A-W, Tetzlaff JM, Gøtzsche PC, Altman DG, Mann H, Berlin J, Dickersin K, Hróbjartsson A, Schulz KF, Parulekar WR, Krleža-Jerić K, Laupacis A, Moher D. SPIRIT 2013 Explanation and Elaboration: Guidance for protocols of clinical trials. BMJ. 2013;346:e7586

		Reporting Item	Page Number
**Administrative information**			
Title	#1	Descriptive title identifying the study design, population, interventions, and, if applicable, trial acronym	1
Trial registration	#2a	Trial identifier and registry name. If not yet registered,	3

For peer review only

		name of intended registry	
Trial registration: data set	#2b	All items from the World Health Organization Trial Registration Data Set	3
Protocol version	#3	Date and version identifier	1
Funding	#4	Sources and types of financial, material, and other support	12
Roles and responsibilities: contributorship	#5a	Names, affiliations, and roles of protocol contributors	11
Roles and responsibilities: sponsor contact information	#5b	Name and contact information for the trial sponsor	12
Roles and responsibilities: sponsor and funder	#5c	Role of study sponsor and funders, if any, in study design; collection, management, analysis, and interpretation of data; writing of the report; and the decision to submit the report for publication, including whether they will have ultimate authority over any of these activities	12
Roles and responsibilities: committees	#5d	Composition, roles, and responsibilities of the coordinating centre, steering committee, endpoint adjudication committee, data management team, and other individuals or groups overseeing the trial, if applicable (see Item 21a for data monitoring committee)	N/A

Introduction

For peer review only

Background and rationale	#6a	Description of research question and justification for undertaking the trial, including summary of relevant studies (published and unpublished) examining benefits and harms for each intervention	5
Background and rationale: choice of comparators	#6b	Explanation for choice of comparators	6
Objectives	#7	Specific objectives or hypotheses	6
Trial design	#8	Description of trial design including type of trial (eg, parallel group, crossover, factorial, single group), allocation ratio, and framework (eg, superiority, equivalence, non-inferiority, exploratory)	6
**Methods: Participants, interventions, and outcomes**			
Study setting	#9	Description of study settings (eg, community clinic, academic hospital) and list of countries where data will be collected. Reference to where list of study sites can be obtained	6
Eligibility criteria	#10	Inclusion and exclusion criteria for participants. If applicable, eligibility criteria for study centres and individuals who will perform the interventions (eg,	7

For peer review only

		surgeons, psychotherapists)	
Interventions: description	#11a	Interventions for each group with sufficient detail to allow replication, including how and when they will be administered	7
Interventions: modifications	#11b	Criteria for discontinuing or modifying allocated interventions for a given trial participant (eg, drug dose change in response to harms, participant request, or improving / worsening disease)	7
Interventions: adherance	#11c	Strategies to improve adherence to intervention protocols, and any procedures for monitoring adherence (eg, drug tablet return; laboratory tests)	7
Interventions: concomitant care	#11d	Relevant concomitant care and interventions that are permitted or prohibited during the trial	N/A
Outcomes	#12	Primary, secondary, and other outcomes, including the specific measurement variable (eg, systolic blood pressure), analysis metric (eg, change from baseline, final value, time to event), method of aggregation (eg, median, proportion), and time point for each outcome. Explanation of the clinical relevance of chosen efficacy and harm outcomes is strongly recommended	8
Participant timeline	#13	Time schedule of enrolment, interventions (including any run-ins and washouts), assessments, and visits for participants. A schematic diagram is highly recommended (see Figure)	8

For peer review only

Sample size	#14	Estimated number of participants needed to achieve study objectives and how it was determined, including clinical and statistical assumptions supporting any sample size calculations	7
Recruitment	#15	Strategies for achieving adequate participant enrolment to reach target sample size	6
**Methods: Assignment of interventions (for controlled trials)**			
Allocation: sequence generation	#16a	Method of generating the allocation sequence (eg, computer-generated random numbers), and list of any factors for stratification. To reduce predictability of a random sequence, details of any planned restriction (eg, blocking) should be provided in a separate document that is unavailable to those who enrol participants or assign interventions	N/A
Allocation concealment mechanism	#16b	Mechanism of implementing the allocation sequence (eg, central telephone; sequentially numbered, opaque, sealed envelopes), describing any steps to conceal the sequence until interventions are assigned	N/A
Allocation: implementation	#16c	Who will generate the allocation sequence, who will enrol participants, and who will assign participants to interventions	N/A

For peer review only

Blinding (masking)	#17a	Who will be blinded after assignment to interventions (eg, trial participants, care providers, outcome assessors, data analysts), and how	N/A
Blinding (masking): emergency unblinding	#17b	If blinded, circumstances under which unblinding is permissible, and procedure for revealing a participant's allocated intervention during the trial	N/A
**Methods: Data collection, management, and analysis**			
Data collection plan	#18a	Plans for assessment and collection of outcome, baseline, and other trial data, including any related processes to promote data quality (eg, duplicate measurements, training of assessors) and a description of study instruments (eg, questionnaires, laboratory tests) along with their reliability and validity, if known. Reference to where data collection forms can be found, if not in the protocol	8
Data collection plan: retention	#18b	Plans to promote participant retention and complete follow-up, including list of any outcome data to be collected for participants who discontinue or deviate from intervention protocols	8
Data management	#19	Plans for data entry, coding, security, and storage, including any related processes to promote data quality (eg, double data entry; range checks for data values).	10

For peer review only

		Reference to where details of data management procedures can be found, if not in the protocol	
Statistics: outcomes	#20a	Statistical methods for analysing primary and secondary outcomes. Reference to where other details of the statistical analysis plan can be found, if not in the protocol	10
Statistics: additional analyses	#20b	Methods for any additional analyses (eg, subgroup and adjusted analyses)	10
Statistics: analysis population and missing data	#20c	Definition of analysis population relating to protocol non-adherence (eg, as randomised analysis), and any statistical methods to handle missing data (eg, multiple imputation)	N/A
**Methods: Monitoring**			
Data monitoring: formal committee	#21a	Composition of data monitoring committee (DMC); summary of its role and reporting structure; statement of whether it is independent from the sponsor and competing interests; and reference to where further details about its charter can be found, if not in the protocol. Alternatively, an explanation of why a DMC is not needed	N/A
Data monitoring: interim analysis	#21b	Description of any interim analyses and stopping guidelines, including who will have access to these interim results and make the final decision to terminate the trial	N/A
Harms	#22	Plans for collecting, assessing, reporting, and managing solicited and spontaneously reported adverse events and other unintended effects of trial interventions or trial	8

For peer review only

		conduct	
Auditing	#23	Frequency and procedures for auditing trial conduct, if any, and whether the process will be independent from investigators and the sponsor	N/A
**Ethics and dissemination**			
Research ethics approval	#24	Plans for seeking research ethics committee / institutional review board (REC / IRB) approval	11
Protocol amendments	#25	Plans for communicating important protocol modifications (eg, changes to eligibility criteria, outcomes, analyses) to relevant parties (eg, investigators, REC / IRBs, trial participants, trial registries, journals, regulators)	N/A
Consent or assent	#26a	Who will obtain informed consent or assent from potential trial participants or authorised surrogates, and how (see Item 32)	6
Consent or assent: ancillary studies	#26b	Additional consent provisions for collection and use of participant data and biological specimens in ancillary studies, if applicable	N/A
Confidentiality	#27	How personal information about potential and enrolled participants will be collected, shared, and maintained in order to protect confidentiality before, during, and after the trial	6
Declaration of	#28	Financial and other competing interests for principal	12

For peer review only

interests		investigators for the overall trial and each study site	
Data access	#29	Statement of who will have access to the final trial dataset, and disclosure of contractual agreements that limit such access for investigators	11
Ancillary and post trial care	#30	Provisions, if any, for ancillary and post-trial care, and for compensation to those who suffer harm from trial participation	N/A
Dissemination policy: trial results	#31a	Plans for investigators and sponsor to communicate trial results to participants, healthcare professionals, the public, and other relevant groups (eg, via publication, reporting in results databases, or other data sharing arrangements), including any publication restrictions	11
Dissemination policy: authorship	#31b	Authorship eligibility guidelines and any intended use of professional writers	N/A
Dissemination policy: reproducible research	#31c	Plans, if any, for granting public access to the full protocol, participant-level dataset, and statistical code	11
**Appendices**			
Informed consent materials	#32	Model consent form and other related documentation given to participants and authorised surrogates	N/A
Biological specimens	#33	Plans for collection, laboratory evaluation, and storage of biological specimens for genetic or molecular analysis in the current trial and for future use in ancillary studies, if applicable	N/A

For peer review only

The SPIRIT Explanation and Elaboration paper is distributed under the terms of the Creative Commons Attribution License CC-BY-NC. This checklist was completed on 20. April 2021 using https://www.goodreports.org/, a tool made by the EQUATOR Network in collaboration with Penelope.ai

For peer review only

BMJ Open

Evaluating the feasibility and acceptability of virtual group exercise for older adults delivered by trained volunteers: the ImPACt study protocol

Journal:	*BMJ Open*
Manuscript ID	bmjopen-2021-052631.R1
Article Type:	Protocol
Date Submitted by the Author:	07-Oct-2021
Complete List of Authors:	Lim, Stephen; University of Southampton, Academic Geriatric Medicine; University of Southampton, NIHR ARC Wessex<br>Meredith, Samantha; University of Southampton, Academic Geriatric Medicine; University of Southampton, NIHR ARC Wessex<br>Agnew, Samantha; The Brendoncare Foundation<br>Clift, Esther; Southern Health NHS Foundation Trust<br>Ibrahim, Kinda; University of Southampton, Academic Geriatric Medicine, Faculty of Medicine; University of Southampton, NIHR ARC Wessex<br>Roberts, Helen; University of Southampton, Academic Geriatric Medicine; University of Southampton, NIHR ARC Wessex
**Primary Subject Heading**:	Geriatric medicine
Secondary Subject Heading:	Qualitative research, Sports and exercise medicine
Keywords:	GERIATRIC MEDICINE, QUALITATIVE RESEARCH, REHABILITATION MEDICINE

SCHOLARONE™
Manuscripts

Title: Evaluating the feasibility and acceptability of virtual group exercise for older adults delivered by trained volunteers: the ImPACt study protocol

Protocol version 1.0

Corresponding author: Dr Stephen Eu Ruen Lim

Email: s.e.lim@soton.ac.uk

Address: Mailpoint 807, Academic Geriatric Medicine, University of Southampton,

Southampton General Hospital, Tremona Road,

Southampton, United Kingdom, SO16 6YD

Tel: 02381206131

Co-authors:

Dr Samantha Jane Meredith; University of Southampton, Southampton, UK

Samantha Agnew; The Brendoncare Foundation, Winchester, UK

Esther Clift; Southern Health NHS Foundation Trust, Southampton, UK

Dr Kinda Ibrahim; University of Southampton, Southampton, UK

Professor Helen C Roberts; University of Southampton, Southampton, UK

Key words:

Exercise, Older adults, volunteer, physical activity

Allowed word count: 4000

Word count: 3911 words

For peer review only

Abstract

Introduction

Physical activity is important for healthy ageing. Despite strong evidence on the benefits of physical activity for health and well-being, physical inactivity remains a significant problem among older adults. This study aims to determine the feasibility and acceptability of implementing an online volunteer-led group exercise for older adults.

Methods

A quasi-experimental mixed methods approach will be used in this study. A training programme will be developed to train volunteers to deliver online group exercises to older adults aged >65 years(n=30). The primary outcome is the feasibility of implementing the intervention. This will be assessed by the number of volunteers recruited, trained, and retained at the end of the study, and the number of exercise sessions delivered and completed by participants. Secondary outcomes include physical activity levels measured using the Community Health Model Activities Program for Seniors(CHAMPS) questionnaire, Barthel Index, EQ-5D-5L as a measure of health-related quality of life, SARC F to determine sarcopenia status, and PRIMSA-7 to determine frailty status. Outcomes will be measured at baseline and at 6 months.

Qualitative interviews will be conducted with volunteers(n=5), older adults(n=10), and family members(n=5) to explore their views on the intervention.

Analysis

Simple descriptive statistics will be used to describe participant characteristics, the feasibility of the study and the impact of the intervention on health outcomes. Parametric(t-test) or non-parametric(Mann-Whitney U test) statistics will be used to analyse continuous variables. Chi-square test will be used for categorical variables. Qualitative data will be analysed using an inductive thematic analysis approach.

Ethics and Dissemination

This study received ethical approval from the University of Southampton Faculty of Medicine Ethics Committee and Research Integrity and Governance committee (ID: 52967.A1). Study findings will be made available to service users, voluntary organisations and other researchers who may be interested in implementing the intervention.

For peer review only - http://bmjopen.bmj.com/site/about/guidelines.xhtml

Trial registration number

Clinicaltrials.gov: NCT04672200

For peer review only

For peer review only - http://bmjopen.bmj.com/site/about/guidelines.xhtml

Article summary

Strengths and limitations of this study

- This is a novel study exploring the use of trained volunteers to deliver online group exercises for older adults.
- It addresses the issue of physical inactivity among older people, a problem which has been exacerbated by the COVID-19 pandemic.
- A mixed methods approach will support the development of a toolkit for future implementation.
- For safety reasons, volunteers will deliver seated exercises in online groups.
- The exclusion of older adults who are not able to provide consent limits the generalisability of this study.

For peer review only

For peer review only - http://bmjopen.bmj.com/site/about/guidelines.xhtml

Introduction

Physical activity is important for healthy ageing. Some of the benefits of regular physical activity for older people include reduction in falls(1), better physical functioning(2), and improvement in well-being(3). The recommended physical activity level for older adults aged 65 years and above is to achieve 150 minutes of moderate physical activity in a week, with 2 days of strength training(4). This is often expressed as 30 minutes of brisk walking or equivalent activity five days a week, although 75 minutes of vigorous intensity activity spread across the week, or a combination of moderate and vigorous activity are also suggested(5).

Despite strong evidence for the benefits of physical activity, physical inactivity remains a significant problem among older adults worldwide(6). Studies in the UK have shown that less than 7% of community-dwelling older adults achieve the recommended weekly physical activity level(7, 8). The COVID-19 pandemic has exacerbated the problem of physical inactivity(9). Rules on social distancing and shielding of vulnerable groups have resulted in increased physical inactivity among older people, with potentially widespread deconditioning.

The rise of the use of technology during the pandemic may provide a solution to address the problem of physical inactivity among older people. Older adults are embracing modern technology more than ever before and are increasingly familiar with the use of devices and online platforms to stay in touch with their family members and friends(10, 11). This presents the opportunity to develop online physical activity interventions for older adults to promote increased physical activity. A review of reviews conducted by McGarrigle et al. found low to moderate evidence that physical activity interventions delivered through the internet and related technologies may be effective in increasing physical activity among older adults in the short term(12). Online group exercises have been shown to be feasible for older adults(13). However, there is little evidence on the use of trained volunteers to lead such exercise groups.

Volunteers play an integral part within many health and social care organisations. They enrich patient experience, help deliver high quality services, and support communities in living healthier lives(14). A report by the King's Fund on volunteering has highlighted that the support volunteers provide can be of particular value to those who rely most heavily on services, such as people with multiple long-term conditions or mental health problems(15). There is increasing evidence to suggest that with proper training, volunteers can take on more direct roles in supporting older adults particularly with nutrition(16) and mobility(17).

For peer review only - http://bmjopen.bmj.com/site/about/guidelines.xhtml

The Southampton Mobility Volunteer study (n=100, mean age 86 years) showed that volunteers can be trained to deliver chair-based exercises to older adults admitted to hospital(17). The intervention was shown to be safe, and acceptable to older adults and healthcare professionals, with evidence of improvement in daily step count among patients who received the intervention. The median age of the volunteers in the study was 32 years (interquartile ratio of 17 -62 years). A systematic review of 12 studies found some evidence suggesting that volunteer-led physical activity interventions that include resistance exercise training, can improve outcomes of community-dwelling older adults including functional status, frailty status and reduction in fear of falls(18). However, knowledge gaps still exist on how best to recruit, train, and retain volunteers to deliver physical activity interventions routinely. This study addresses this knowledge gap by exploring facilitators and barriers to the implementation of volunteer-led group exercises for older adults attending social clubs.

Older adults who volunteer to lead physical activity interventions also benefit from their volunteering, with one study showing evidence of improved physical activity levels(19). Older volunteers also benefit from the social interaction that volunteering brings, with reduced feelings of loneliness and having a sense of achievement(20). The use of trained volunteers to deliver online physical activity interventions remains an under-researched area and this study addresses the important knowledge gap on the feasibility and acceptability of such interventions(21).

Aims

This study aims to determine the feasibility and acceptability of training volunteers to deliver online group exercises to community-dwelling older adults. The specific objectives of this study are to:

1. Develop a training programme for volunteers to lead online group exercises
2. Determine the feasibility of recruiting, training and retaining volunteers to deliver the online intervention
3. Determine the acceptability of the online intervention to volunteers, older adults, and their carers
4. Explore barriers and facilitators to the implementation of the online intervention

For peer review only - http://bmjopen.bmj.com/site/about/guidelines.xhtml

Methods and Analysis

Study design

A quasi-experimental mixed methods approach will be used to determine the feasibility and acceptability of implementing online volunteer-led exercise sessions in community clubs. The study will be conducted at community-clubs focussed on promoting social interaction among older people. Feasibility studies are used to determine whether an intervention is appropriate for further evaluation, to determine sample sizes for controlled trials and to assess whether the ideas and findings can be shaped to be relevant and sustainable. In this study, the feasibility of training volunteers to promote increased physical activity among community-dwelling older adults will be assessed. The acceptability of the intervention will be examined through qualitative interviews to explore the views and experiences of volunteers, older adults participating in the study, and their carers or family members. The impact of the intervention on physical activity levels and functional outcomes will also be measured. This will help inform the sample size calculation for a future effectiveness trial.

Participant recruitment

Volunteer recruitment

The research team will be invited by club staff to attend the virtual clubs to introduce the study. Club volunteers and members who are interested in leading the group exercise will indicate their interest to staff members or approach the research team directly. To lead the group exercise, volunteers must be physically able to perform the exercises themselves and be able to provide written consent. This will be assessed at the training sessions by the trainer (SM).

The participant information sheet and the consent form will be sent through the post. Club members are invited to contact the research team for further discussions about the study. Once the research team has received the signed consent form, volunteers will be invited to participate in the online training. To deliver online group exercise to 30 exercise participants, it is estimated that at least 12 volunteers will be needed to lead 6 online groups. Volunteers will be paired up to lead and deliver the intervention.

Volunteer training

For peer review only - http://bmjopen.bmj.com/site/about/guidelines.xhtml

The volunteer training programme will be led by a member of the research team with a background in sport and exercise science. The training programme will be developed based on clinical expertise from therapists and current evidence including experience from a recently completed hospital-based study(17). The training session will include key aspects such as personal safety, safety of participants, and the exercise protocol. Examples of personal safety include ensuring appropriate clothing and footwear, having good ventilation in the room, staying well-hydrated, and adopting a correct posture whilst performing the exercises. Volunteers will be trained to coach the participants, ensuring that the exercises are performed safely and effectively to prevent injuries. They will receive training on how to document adverse events that occur during the exercise classes. They will also be given guidance on how to set up their digital devices. Each volunteer will receive two group training sessions, lasting an hour each, and further one to one sessions will be offered to volunteers who wish to have further training. Volunteers will receive a training booklet containing the exercises and safety instructions, as well as an exercise video to support their training. Competency assessments based on a checklist will be conducted by the research team to ensure that volunteers are competent in delivering the exercises online (supplement 1). Throughout the study period, fidelity checks will be conducted to ensure that the volunteers are delivering high quality exercise sessions. The fidelity checks will comprise assessment of the volunteer exercise delivery including the safe and effective demonstration and instruction of the warm-up, strength exercise, and cool down components of the exercises. A member of the research team will attend the group online sessions as an observer to conduct the checks. Volunteers who require further support in delivering the exercises will be offered additional coaching by the research team to ensure that the exercises are delivered correctly.

Regular meetings for the volunteers will be held throughout the study period to provide them with ongoing support from the research team and the organisation running the online clubs. The group meetings will provide an avenue for volunteers to discuss and share their experiences, ask questions, and provide suggestions to improve the intervention.

Exercise participant recruitment

Club members who are interested in participating in the online group exercise will indicate their interest to club staff or approach the research team directly. Participant information sheet and consent forms will be posted to club members.

The inclusion criteria for the group exercise participants are: adults aged 65 years and older, able to walk with or without a walking aid, and able to provide written consent (Table 1). Participants who

For peer review only - http://bmjopen.bmj.com/site/about/guidelines.xhtml

For peer review only

are actively participating in other online exercise classes will be excluded. The aim is to recruit 30 older adults attending social clubs who do not engage in regular exercise.

Intervention

The intervention consists of a once weekly volunteer-led online group home-based exercise, for a duration of 30 minutes. The online group will be led by volunteers and hosted on the digital platform Zoom(22). To comply with the social club's online insurance policy and to minimise the risk of injury to participants, the intervention will only include seated exercises. The seated exercises will focus on strengthening upper and lower limbs and enhancing whole body range of motion and flexibility, tailored specifically to meet the needs of older adults through considering appropriate FITT (frequency, intensity, time, type) principles and exercise adaptations (e.g., in response to joint pain and discomfort). Participants will be encouraged by the volunteers to progress by increasing repetitions and to gently improve range of motion. No supplementary equipment is needed for the online exercises. In addition to the group exercise sessions, participants will also be encouraged to increase mobility and physical activity levels through use of a home activity booklet, including a physical activity diary. The exercise sessions will be piloted in one club before being extended to other clubs.

Outcomes measures

Volunteer characteristics such as age, gender, employment status, previous volunteering experience, and experience in delivering group exercises will be recorded. Participant characteristics including age, gender, domicile status, body mass index, co-morbidities, cognition (telephone mini-mental state examination) and number of medications will be recorded.

Primary outcomes

The primary outcome of this study is the feasibility of implementing volunteer-led online group exercise for older adults. This will be assessed by determining:

1. the number of volunteers recruited, trained, and retained at the end of the study,
2. the number of physical activity sessions delivered and completed by participants (adherence), and
3. the fidelity of the exercise intervention delivered by volunteers.

For peer review only - http://bmjopen.bmj.com/site/about/guidelines.xhtml

Secondary outcomes

The secondary outcome measures include measurement of physical activity levels and performance of activities of daily living. Physical activity levels will be measured using the Community Health Model Activities Program for Seniors (CHAMPS) questionnaire(23). The questionnaire assesses self-reported weekly frequency and duration of various physical activities typically undertaken by older adults, and is a validated measure of physical activity for older people(24). Performance of activities of daily living (ADL) will be measured using the Barthel Index(25). The Barthel Index measures ADL including transfers, walking, stairs, toilet use, dressing, feeding, bladder control, bowel control, grooming and bathing, to give a maximum total score of 100. It is a validated measure of ADL that is recommended for use in research among older people(26). The EQ-5D-5L measure developed by the EuroQol group will be used to determine health-related quality of life(27). The measure comprises five dimensions: mobility, self-care, usual activities, pain/discomfort, and anxiety/ depression. Each dimension is given a 1- digit score and the digits for the five dimensions are combined unto a 5-digit number that describes the patient's health state. The measure is commonly used in research among older people(28, 29). The SARC-F questionnaire will be used as a measure to assess for sarcopenia(30). According to recent guidelines from the European Working Group on Sarcopenia in Older People, SARC-F is the recommended tool for sarcopenia case finding (31). It comprises five components: strength, assistance in walking, rise from a chair, climbing stairs, and falls. The scores range from 0 to 10, with a score of equal to or greater than four being predictive of sarcopenia and poor outcome. PRISMA-7 will be used to characterise participants' frailty status(32). The questionnaire contains seven items with a positive score of three or more indicating frailty. The PRISMA-7 tool has been shown to have high accuracy in identifying frail older adults in the community setting(33).

These outcome measures will be recorded at baseline, using remote assessment formats (e.g., telephone-based) and repeated at 6 months. Cost analysis will be conducted to determine the cost of implementing this intervention. The analysis will include the cost of training the volunteers, taking into consideration the time needed to deliver the group training sessions and the individual competency assessments, and the provision of training materials.

Safety and adverse events

To determine the safety of the intervention, adverse events that occur as a result of the intervention will be recorded. Any injuries or symptoms developed directly as a result of the exercises will be recorded as an adverse event. Volunteers will be trained to document any adverse events and report

For peer review only

For peer review only - http://bmjopen.bmj.com/site/about/guidelines.xhtml

it back to the research team. The clubs also have well established procedures for responding to incidents and accidents including access to emergency contacts of participants.

Acceptability of the intervention

Interviews will be conducted with older adults, their family members or carers, volunteers, and those involved in recruiting participants and training volunteers, to determine the acceptability of the training and the intervention and to explore barriers and enablers to the implementation of the intervention. Interviews will be semi-structured to help guide conversation whilst allowing participants the flexibility to elaborate and reflect on any meaningful experiences(34). The interviews will be conducted by a member of the research team (SM) who is experienced in qualitative interviews. The interviews will be conducted within the first 2 months of the exercise groups to understand how the intervention is being delivered and whether any modifications to the intervention are needed. Further interviews will be conducted towards the end of the study period (at 6 months) to understand stakeholders' lived experience with the intervention and to explore barriers and facilitators for engagement and delivery of the intervention. This will ensure that participants' views are captured during the earlier stages of the intervention, and when the groups are well-established.

Older adults will be selected by purposive sampling to share their thoughts and views regarding the implementation of the volunteer-led physical activity session(35). To ensure that a wide range of views are included in the interviews, participants will be selected to include male and female participants, different clubs, and a representative age range. The aim is to interview at least two participants per club, with a total number of 10 participants. Family members and carers (n=5) of the exercise participants will also be invited to participate in the interviews. Their views and experience will provide valuable insight into the acceptability of the intervention and the potential barriers and enablers for a future wider implementation. All interviews will be conducted via telephone or online (e.g., Zoom, Skype) depending upon participant preference.

Semi-structured interviews will also be conducted with volunteers leading the intervention. Discussions among volunteers are likely to generate a wider perspective of the implementation process and may provide insight into the group narrative on their experiences in delivering the intervention and their interaction with the research participants(36, 37). The aim is to interview at least one volunteer from each club, with a total number of five volunteers.

The semi-structured interviews will consist of several key open-ended questions that help define the areas explored but allow the interviewer or interviewee to expand and diverge with the aim of

For peer review only - http://bmjopen.bmj.com/site/about/guidelines.xhtml

pursuing or developing an idea with more depth. The interview schedules will be underpinned by Normalisation Process Theory (NPT)(38). The interviews will be audio-recorded for data collection purposes.

Normalisation Process Theory

The NPT provides a set of sociological tools to understand and explain the social processes through which new or modified practice of thinking, enacting, and organizing work are operationalized in healthcare and other institutional settings(38). The use of an implementation theory is useful as it helps researchers identify, describe and explain important elements of the implementation process(39). NPT was chosen as the framework that underpinned this study as it was systematic in its approach, easy to understand and provided a structured framework to evaluate the implementation process of this study. The online toolkit available for this implementation theory also made the application of NPT convenient and practical(40). The interview schedules were developed based on the four components of NPT:

I. Coherence: meaning and sense-making.
II. Cognitive participation: this explores the likelihood of all participants being committed and engaged with the intervention.
III. Collective action: the consideration of the work that participants do to make the intervention function.
IV. Reflexive monitoring: which involves participants appraising or reflecting on the intervention.

The questions will be framed to explore each component of the NPT in greater detail, with the aim to determine factors that facilitated or inhibited these four key processes which are essential for successful implementation and adoption of the intervention. The first section of the interview guide will focus on interviewees' views on the relevance and importance of physical activity in general, and in particular, the online exercises. Next, participants' views on factors that encouraged or prevented them from being committed and engaged with the online group exercise will be explored. Volunteers, staff members, older adults, and their carers' experience of the exercise group and how the stakeholders interacted with each other will then be explored to better understand the impact of the group dynamics on the intervention. Interviewees will then be encouraged to reflect on what worked well with the intervention and explore aspects within the intervention that could be improved or require modification.

For peer review only - http://bmjopen.bmj.com/site/about/guidelines.xhtml

Data analysis plan

Quantitative data analysis

Data collected will be double entered into a secured database for analysis. Statistical analysis will be conducted using the statistical software SPSS Statistics 25. Descriptive statistics -median (IQR); mean (SD); number (%) – will be used to analyse the numbers of volunteers recruited, trained, and retained, as well as the type and extent of progression of the seated exercise, and duration of their activity, and patients' adherence to the intervention. Analysis of the above outcome measures will be used to develop an assessment of the feasibility of delivering this intervention. Adherence to the intervention and reasons for non-adherence will be reported.

Outcome measures recorded at baseline will be compared to measurement at 6 months to determine if the PA intervention had an impact on physical activity levels measured by CHAMPS, functional outcomes including performance in activities of daily living (Barthel Index), quality of life (EQ-5D-5L l), frailty status (PRISMA 7) and sarcopenia status (SARC-F). The distribution of each outcome measure will be assessed for normality. Depending on the skew of the data, parametric (t-test) or non-parametric (Mann-Whitney U test) statistics will be used for continuous variables. Chi-square test will be used for categorical variables.

Qualitative data analysis

Data collected from the interviews will be transcribed verbatim and analysed with an inductive thematic analysis approach(41). This approach includes: Phase 1 – familiarising with the data, Phase 2 – generating initial codes, Phase 3 – searching for themes, Phase 4 – reviewing themes, Phase 5 – defining and naming themes, and Phase 6 – producing the report. Analysis of qualitative data will be conducted using NVivo. A sample of transcribed text will be read and coded separately and then together by two researchers to create a coding manual that applies to the entire set of transcripts. The codes will be analysed to generate concepts and ideas to determine the acceptability of the intervention, and to identify facilitators and barriers to the implementation process. The codes act as tags or labels to help catalogue key concepts embedded within the raw data. From the codes, themes will be developed to reflect the views and experiences of the community-dwelling older adults and volunteers regarding the online physical activity intervention.

A toolkit that will support a wider implementation of the intervention will be developed. It will be a written manual which contains the training programme, exercises, competency checklists, and information on how best to implement the intervention, taking into consideration the barriers and facilitators determined through the qualitative interviews.

For peer review only - http://bmjopen.bmj.com/site/about/guidelines.xhtml

For peer review only

Patient and public involvement

PPI involvement was sought in the development of this protocol. Visits were made to two clubs and discussions were held among club volunteers and club attendees about this project. Their views on what type of activities and the frequency of the activities were explored. Club attendees were also asked about what outcomes were important to them, which will be included in the study.

SA who is a co-applicant in this study, will also represent the views of club members and volunteers. She is the head of clubs for the organisation and is experienced in coordinating clubs. She has an overview on how the clubs are conducted and her input on the practical aspects of implementing the intervention will be invaluable. Her practical experience gained through interaction with club members and volunteers will help ensure that their views are represented.

Two additional PPI representatives will be invited to be part of the study management group led by the PPI lead for the Ageing and Dementia theme within the Wessex ARC. This group will aim to meet up quarterly to discuss the study progress and will be involved in the design of the research, discussion of the results and dissemination of research findings. PPI has also been involved in the preparation of public-facing materials and study documents.

Strengths and limitations of the study

This study explores a novel approach of training volunteers to deliver online group exercise to older adults. Application of a mixed methods approach in this study will help determine the feasibility of the intervention and explore barriers and facilitators to a successful implementation of the intervention. However, a limitation of the study is the lack of a control group. Recruitment of older adults will be through established social clubs, in which recruiting some older adults and excluding others from participating in the exercise groups would be a challenge. Additionally, as this is a feasibility study, we will use a range of social clubs of varying sizes, and client groups with different age ranges and physical abilities, to explore different approaches to the intervention rather than including a control group. Nevertheless, this study has been designed to assess the feasibility and acceptability of the intervention with a view to informing the development of a toolkit to support the wider implementation of the intervention and a future controlled experimental design.

For peer review only - http://bmjopen.bmj.com/site/about/guidelines.xhtml

Ethics and dissemination

Ethical and safety considerations

This study has received ethical approval from the University of Southampton Faculty of Medicine Ethics Committee and Research Integrity and Governance committee (ID: 52967.A1) Data will be stored on university repository PURE and will be made available to other researchers on request.

Dissemination plan

Findings from this study will be published in peer-reviewed journals and presented at national and international scientific platforms. A toolkit will be developed to support future implementation plans.

Author Contributions

SL, EC, SA, KI, and HCR were involved in the conception and trial design. SM will lead the volunteer training and be involved in data collection, and analysis. All authors contributed in the preparation of this article.

Funding statement

The National Institute for Health Research (NIHR) Applied Research Collaboration Wessex funded this research (Award number: not applicable). The views expressed are those of the authors and not necessarily those of the NHS, the NIHR or the Department of Health. SL, SM, KI, and HCR received support from the NIHR Applied Research Collaboration (ARC) Wessex and the University of Southampton. HCR received support from the NIHR Southampton Biomedical Research Centre. The University of Southampton NIHR Academic Clinical Lectureship scheme supported SL (Award number: CL2018-26-002).

Competing Interests

The authors have no competing interests to declare.

Table 1 Inclusion and exclusion criteria

Inclusion Criteria
Older adults aged > 65 years<br>Able to walk, with or without a walking aid<br>Able to provide written consent
Exclusion Criteria

For peer review only

Actively participating in other online exercise classes

For peer review only

For peer review only - http://bmjopen.bmj.com/site/about/guidelines.xhtml

References

1. Sherrington C, Fairhall N, Kwok W, Wallbank G, Tiedemann A, Michaleff ZA, et al. Evidence on physical activity and falls prevention for people aged 65+ years: systematic review to inform the WHO guidelines on physical activity and sedentary behaviour. International Journal of Behavioral Nutrition and Physical Activity. 2020;17(1):144.
2. Halaweh H, Willén C, Svantesson U. Association between physical activity and physical functioning in community-dwelling older adults. European Journal of Physiotherapy. 2017;19(1):40-7.
3. Fox KR, Stathi A, McKenna J, Davis MG. Physical activity and mental well-being in older people participating in the Better Ageing Project. European Journal of Applied Physiology. 2007;100(5):591-602.
4. Foster C, Reilly J, Jago R, Murphy M, Skelton D, Cooper A, et al. UK Chief Medical Officers' Physical Activity Guidelines. GOV.UK: UK Department of Health and Social Care 2019 07/09/2019.
5. Sparling PB, Howard BJ, Dunstan DW, Owen N. Recommendations for physical activity in older adults. BMJ. 2015;350(jan20 6):h100.
6. Cunningham C, R OS, Caserotti P, Tully MA. Consequences of physical inactivity in older adults: A systematic review of reviews and meta-analyses. Scandinavian journal of medicine & science in sports. 2020;30(5):816-27.
7. Harris TJ, Owen CG, Victor CR, Adams R, Cook DG. What factors are associated with physical activity in older people, assessed objectively by accelerometry? British journal of sports medicine. 2009;43(6):442-50.
8. Jefferis BJ, Sartini C, Lee IM, Choi M, Amuzu A, Gutierrez C, et al. Adherence to physical activity guidelines in older adults, using objectively measured physical activity in a population-based study. BMC public health. 2014;14(1):382-.
9. Wider impacts of COVID-19 on physical activity, deconditioning and falls in older adults. Public Health England. Available from: https://www.gov.uk/government/publications/covid-19-wider-impacts-on-people-aged-65-and-over. Last accessed: 05/10/2021
10. Marston HR, Genoe R, Freeman S, Kulczycki C, Musselwhite C. Older Adults' Perceptions of ICT: Main Findings from the Technology In Later Life (TILL) Study. Healthcare (Basel). 2019;7(3):86.
11. Barbosa Neves B, Franz R, Judges R, Beermann C, Baecker R. Can Digital Technology Enhance Social Connectedness Among Older Adults? A Feasibility Study. Journal of applied gerontology : the official journal of the Southern Gerontological Society. 2019;38(1):49-72.
12. McGarrigle L, Todd C. Promotion of Physical Activity in Older People Using mHealth and eHealth Technologies: Rapid Review of Reviews. J Med Internet Res. 2020;22(12):e22201.
13. Baez M, Khaghani Far I, Ibarra F, Ferron M, Didino D, Casati F. Effects of online group exercises for older adults on physical, psychological and social wellbeing: a randomized pilot trial. PeerJ. 2017;5:e3150.
14. Mundle C, Naylor C, Buck D. Volunteering in health and care in England: A summary of key literature. 2012. Available from: https://www.kingsfund.org.uk/sites/default/files/field/field_related_document/volunteering-in-health-literature-review-kingsfund-mar13.pdf. Last accessed: 05/10/2021
15. Naylor C, Mundle C, Weaks L, Buck D. Volunteering in health and care: Securing a sustainable future 2013 [28/07/2015]. Available from: http://www.kingsfund.org.uk/publications/volunteering-health-and-care. Last accessed: 05/10/2021
16. Roberts HC, De Wet S, Porter K, Rood G, Diaper N, Robison J, et al. The feasibility and acceptability of training volunteer mealtime assistants to help older acute hospital inpatients: the Southampton Mealtime Assistance Study. Journal of clinical nursing. 2014;23(21-22):3240-9.

For peer review only - http://bmjopen.bmj.com/site/about/guidelines.xhtml

17. Lim S, Ibrahim K, Dodds R, Purkis A, Baxter M, Rogers A, et al. Physical activity in hospitalised older people: the feasibility and acceptability of a volunteer-led mobility intervention in the SoMoVe study. Age and Ageing. 2020;49(2):283-91.
18. Lim SER, Cox NJ, Tan QY, Ibrahim K, Roberts HC. Volunteer-led physical activity interventions to improve health outcomes for community-dwelling older people: a systematic review. Aging Clin Exp Res. 2021;33(4):843-53.
19. Tan EJ, Xue Q-L, Li T, Carlson MC, Fried LP. Volunteering: a physical activity intervention for older adults--The Experience Corps program in Baltimore. J Urban Health. 2006;83(5):954-69.
20. Withall J, Thompson JL, Fox KR, Davis M, Gray S, Koning Jd, et al. Participant and Public Involvement in Refining a Peer-Volunteering Active Aging Intervention: Project ACE (Active, Connected, Engaged). Gerontologist. 2018;58(2):362-75.
21. Goehner A, Kricheldorff C, Bitzer EM. Trained volunteers to support chronically ill, multimorbid elderly between hospital and domesticity - a systematic review of one-on-one-intervention types, effects, and underlying training concepts. BMC Geriatr. 2019;19(1):126.
22. Zoom [Available from: https://zoom.us/.
23. Hekler EB, Buman MP, Haskell WL, Conway TL, Cain KL, Sallis JF, et al. Reliability and validity of CHAMPS self-reported sedentary-to-vigorous intensity physical activity in older adults. J Phys Act Health. 2012;9(2):225-36.
24. Falck RS, McDonald SM, Beets MW, Brazendale K, Liu-Ambrose T. Measurement of physical activity in older adult interventions: a systematic review. British Journal of Sports Medicine. 2015.
25. Wade DT, Collin C. The Barthel ADL Index: A standard measure of physical disability? International Disability Studies. 1988;10(2):64-7.
26. Hopman-Rock M, van Hirtum H, de Vreede P, Freiberger E. Activities of daily living in older community-dwelling persons: a systematic review of psychometric properties of instruments. Aging Clinical and Experimental Research. 2019;31(7):917-25.
27. Rabin R, Charro Fd. EQ-SD: a measure of health status from the EuroQol Group. Annals of Medicine. 2001;33(5):337-43.
28. Pérez-Ros P, Vila-Candel R, Martin-Utrilla S, Martínez-Arnau FM. Health-Related Quality of Life in Community-Dwelling Older People with Cognitive Impairment: EQ-5D-3L Measurement Properties. Journal of Alzheimer's disease : JAD. 2020;77(4):1523-32.
29. Hulme C, Long AF, Kneafsey R, Reid G. Using the EQ-5D to assess health-related quality of life in older people. Age Ageing. 2004;33(5):504-7.
30. Ida S, Kaneko R, Murata K. SARC-F for Screening of Sarcopenia Among Older Adults: A Meta-analysis of Screening Test Accuracy. Journal of the American Medical Directors Association. 2018;19(8):685-9.
31. Cruz-Jentoft AJ, Bahat G, Bauer J, Boirie Y, Bruyere O, Cederholm T, et al. Sarcopenia: revised European consensus on definition and diagnosis. Age Ageing. 2019;48(1):16-31.
32. Raîche M, Hébert R, Dubois M-F. PRISMA-7: A case-finding tool to identify older adults with moderate to severe disabilities. Archives of Gerontology and Geriatrics. 2008;47(1):9-18.
33. Hoogendijk EO, van der Horst HE, Deeg DJH, Frijters DHM, Prins BAH, Jansen APD, et al. The identification of frail older adults in primary care: comparing the accuracy of five simple instruments. Age and Ageing. 2012;42(2):262-5.
34. Gill P, Stewart K, Treasure E, Chadwick B. Methods of data collection in qualitative research: interviews and focus groups. Br Dent J. 2008;204(6):291-5.
35. Palinkas LA, Horwitz SM, Green CA, Wisdom JP, Duan N, Hoagwood K. Purposeful sampling for qualitative data collection and analysis in mixed method implementation research. Administration and policy in mental health. 2015;42(5):533-44.
36. Guest G, Namey E, Taylor J, Eley N, McKenna K. Comparing focus groups and individual interviews: findings from a randomized study. International Journal of Social Research Methodology. 2017;20(6):693-708.

For peer review only - http://bmjopen.bmj.com/site/about/guidelines.xhtml

37. Bloor M, Frankland J, Thomas M, Robson K. Focus groups in social research. London: Sage Publications; 2001.
38. May CR, Mair F, Finch T, MacFarlane A, Dowrick C, Treweek S, et al. Development of a theory of implementation and integration: Normalization Process Theory. Implementation science : IS. 2009;4(1):29.
39. May C. Towards a general theory of implementation. Implementation Science. 2013;8(1):18.
40. Normalization Process Theory Toolkit [Available from: http://www.normalizationprocess.org/npt-toolkit/. Last accessed: 05/10/2021]
41. Braun V, Clarke V. Using thematic analysis in psychology. Qualitative Research in Psychology. 2006;3:77-101.

For peer review only

For peer review only - http://bmjopen.bmj.com/site/about/guidelines.xhtml

ImPACt Volunteer Competencies

These competencies have been developed to enhance club members care during online exercise sessions. They have been broken down into separate competencies to ensure you feel equipped to work with older adults to promote their activity, whilst maintaining the individuals and your own safety. Each box should be dated and signed by yourself and the supporting therapist or researcher.

Name: **Date started:** **Date completed:**

Skill / Knowledge	Criteria	Training given	Skill practiced with supervision on peers	Skill practiced with supervision on patient	Skill completed (signed by therapist / researcher)
Personal safety awareness	Wear appropriate clothing and footwear to be comfortable when exercising.				
	Ensure your exercise space is set up safely, including a sturdy chair, and the space is free of obstacles.				
	Ensure you are well enough to exercise and know when exercise should be avoided.				
Basic patient safety	Demonstrate what to do if a participant feels unwell. Stop exercise. Participants should not exercise if they have a fever or any new or worsening symptoms.				
	If participants have symptoms indicative of an emergency situation e.g., chest pain, dizziness, or shortness of breath that does not go away upon cessation of exercise, then call 999.				
	CALL FOR HELP IF YOU FEEL IT IS AN EMERGENCY<br>If there is an emergency situation alert a staff member, who will support you.				

For peer review only

	Staff members will have access to participants emergency contact info.				
	Demonstrate what to do if a participant experiences pain during exercise. Stop and review the participants position and technique. Offer an alternative exercise if pain persists, or suggest they miss out the exercise and rest. Advise they seek advice from their GP if pain persists.				
Pre exercise checks	Demonstrate proficiency with online set up and use of an online platform (e.g., Zoom).				
	Introduction to participants / group. Explain role and overview of exercise session.				
	Determine participants readiness and safety to exercise. How are you? How are you feeling?				
	Ensure participants have a safe exercise set-up, including a sturdy chair, water nearby for hydration, and the room is clear of obstacles.				
	Ensure patient has appropriate clothing and footwear to exercise.				
Prescribed exercises	To demonstrate an understanding of the main components of an exercise session, including warm up, conditioning, and cool down.				

For peer review only

	To prompt and demonstrate correct technique for lower limb chair exercises.				
	To prompt and demonstrate correct technique for upper limb chair exercises.				
	To demonstrate verbal coaching proficiency, including clear feedback on exercise technique and rationale to participants.				
	To demonstrate ability to encourage and motivate participants to exercise.				
	To demonstrate understanding of exercise progressions.				
Documentation	Proficiency completing participant registers.				
	Proficiency completing adverse events log.				

For peer review only

Reporting checklist for protocol of a clinical trial.

Based on the SPIRIT guidelines.

Instructions to authors

Complete this checklist by entering the page numbers from your manuscript where readers will find each of the items listed below.

Your article may not currently address all the items on the checklist. Please modify your text to include the missing information. If you are certain that an item does not apply, please write "n/a" and provide a short explanation.

Upload your completed checklist as an extra file when you submit to a journal.

In your methods section, say that you used the SPIRITreporting guidelines, and cite them as:

Chan A-W, Tetzlaff JM, Gøtzsche PC, Altman DG, Mann H, Berlin J, Dickersin K, Hróbjartsson A, Schulz KF, Parulekar WR, Krleža-Jerić K, Laupacis A, Moher D. SPIRIT 2013 Explanation and Elaboration: Guidance for protocols of clinical trials. BMJ. 2013;346:e7586

		Reporting Item	Page Number
**Administrative information**			
Title	#1	Descriptive title identifying the study design, population, interventions, and, if applicable, trial acronym	1
Trial registration	#2a	Trial identifier and registry name. If not yet registered,	3

For peer review only

		name of intended registry	
Trial registration: data set	#2b	All items from the World Health Organization Trial Registration Data Set	3
Protocol version	#3	Date and version identifier	1
Funding	#4	Sources and types of financial, material, and other support	12
Roles and responsibilities: contributorship	#5a	Names, affiliations, and roles of protocol contributors	11
Roles and responsibilities: sponsor contact information	#5b	Name and contact information for the trial sponsor	12
Roles and responsibilities: sponsor and funder	#5c	Role of study sponsor and funders, if any, in study design; collection, management, analysis, and interpretation of data; writing of the report; and the decision to submit the report for publication, including whether they will have ultimate authority over any of these activities	12
Roles and responsibilities: committees	#5d	Composition, roles, and responsibilities of the coordinating centre, steering committee, endpoint adjudication committee, data management team, and other individuals or groups overseeing the trial, if applicable (see Item 21a for data monitoring committee)	N/A

Introduction

For peer review only

Background and rationale	#6a	Description of research question and justification for undertaking the trial, including summary of relevant studies (published and unpublished) examining benefits and harms for each intervention	5
Background and rationale: choice of comparators	#6b	Explanation for choice of comparators	6
Objectives	#7	Specific objectives or hypotheses	6
Trial design	#8	Description of trial design including type of trial (eg, parallel group, crossover, factorial, single group), allocation ratio, and framework (eg, superiority, equivalence, non-inferiority, exploratory)	6
**Methods: Participants, interventions, and outcomes**			
Study setting	#9	Description of study settings (eg, community clinic, academic hospital) and list of countries where data will be collected. Reference to where list of study sites can be obtained	6
Eligibility criteria	#10	Inclusion and exclusion criteria for participants. If applicable, eligibility criteria for study centres and individuals who will perform the interventions (eg,	7

For peer review only

		surgeons, psychotherapists)	
Interventions: description	#11a	Interventions for each group with sufficient detail to allow replication, including how and when they will be administered	7
Interventions: modifications	#11b	Criteria for discontinuing or modifying allocated interventions for a given trial participant (eg, drug dose change in response to harms, participant request, or improving / worsening disease)	7
Interventions: adherance	#11c	Strategies to improve adherence to intervention protocols, and any procedures for monitoring adherence (eg, drug tablet return; laboratory tests)	7
Interventions: concomitant care	#11d	Relevant concomitant care and interventions that are permitted or prohibited during the trial	N/A
Outcomes	#12	Primary, secondary, and other outcomes, including the specific measurement variable (eg, systolic blood pressure), analysis metric (eg, change from baseline, final value, time to event), method of aggregation (eg, median, proportion), and time point for each outcome. Explanation of the clinical relevance of chosen efficacy and harm outcomes is strongly recommended	8
Participant timeline	#13	Time schedule of enrolment, interventions (including any run-ins and washouts), assessments, and visits for participants. A schematic diagram is highly recommended (see Figure)	8

For peer review only

Sample size	#14	Estimated number of participants needed to achieve study objectives and how it was determined, including clinical and statistical assumptions supporting any sample size calculations	7
Recruitment	#15	Strategies for achieving adequate participant enrolment to reach target sample size	6
**Methods: Assignment of interventions (for controlled trials)**			
Allocation: sequence generation	#16a	Method of generating the allocation sequence (eg, computer-generated random numbers), and list of any factors for stratification. To reduce predictability of a random sequence, details of any planned restriction (eg, blocking) should be provided in a separate document that is unavailable to those who enrol participants or assign interventions	N/A
Allocation concealment mechanism	#16b	Mechanism of implementing the allocation sequence (eg, central telephone; sequentially numbered, opaque, sealed envelopes), describing any steps to conceal the sequence until interventions are assigned	N/A
Allocation: implementation	#16c	Who will generate the allocation sequence, who will enrol participants, and who will assign participants to interventions	N/A

For peer review only

Blinding (masking)	#17a	Who will be blinded after assignment to interventions (eg, trial participants, care providers, outcome assessors, data analysts), and how	N/A
Blinding (masking): emergency unblinding	#17b	If blinded, circumstances under which unblinding is permissible, and procedure for revealing a participant's allocated intervention during the trial	N/A
**Methods: Data collection, management, and analysis**			
Data collection plan	#18a	Plans for assessment and collection of outcome, baseline, and other trial data, including any related processes to promote data quality (eg, duplicate measurements, training of assessors) and a description of study instruments (eg, questionnaires, laboratory tests) along with their reliability and validity, if known. Reference to where data collection forms can be found, if not in the protocol	8
Data collection plan: retention	#18b	Plans to promote participant retention and complete follow-up, including list of any outcome data to be collected for participants who discontinue or deviate from intervention protocols	8
Data management	#19	Plans for data entry, coding, security, and storage, including any related processes to promote data quality (eg, double data entry; range checks for data values).	10

For peer review only

		Reference to where details of data management procedures can be found, if not in the protocol	
Statistics: outcomes	#20a	Statistical methods for analysing primary and secondary outcomes. Reference to where other details of the statistical analysis plan can be found, if not in the protocol	10
Statistics: additional analyses	#20b	Methods for any additional analyses (eg, subgroup and adjusted analyses)	10
Statistics: analysis population and missing data	#20c	Definition of analysis population relating to protocol non-adherence (eg, as randomised analysis), and any statistical methods to handle missing data (eg, multiple imputation)	N/A
**Methods: Monitoring**			
Data monitoring: formal committee	#21a	Composition of data monitoring committee (DMC); summary of its role and reporting structure; statement of whether it is independent from the sponsor and competing interests; and reference to where further details about its charter can be found, if not in the protocol. Alternatively, an explanation of why a DMC is not needed	N/A
Data monitoring: interim analysis	#21b	Description of any interim analyses and stopping guidelines, including who will have access to these interim results and make the final decision to terminate the trial	N/A
Harms	#22	Plans for collecting, assessing, reporting, and managing solicited and spontaneously reported adverse events and other unintended effects of trial interventions or trial	8

For peer review only

		conduct	
Auditing	#23	Frequency and procedures for auditing trial conduct, if any, and whether the process will be independent from investigators and the sponsor	N/A
**Ethics and dissemination**			
Research ethics approval	#24	Plans for seeking research ethics committee / institutional review board (REC / IRB) approval	11
Protocol amendments	#25	Plans for communicating important protocol modifications (eg, changes to eligibility criteria, outcomes, analyses) to relevant parties (eg, investigators, REC / IRBs, trial participants, trial registries, journals, regulators)	N/A
Consent or assent	#26a	Who will obtain informed consent or assent from potential trial participants or authorised surrogates, and how (see Item 32)	6
Consent or assent: ancillary studies	#26b	Additional consent provisions for collection and use of participant data and biological specimens in ancillary studies, if applicable	N/A
Confidentiality	#27	How personal information about potential and enrolled participants will be collected, shared, and maintained in order to protect confidentiality before, during, and after the trial	6
Declaration of	#28	Financial and other competing interests for principal	12

For peer review only

interests		investigators for the overall trial and each study site	
Data access	#29	Statement of who will have access to the final trial dataset, and disclosure of contractual agreements that limit such access for investigators	11
Ancillary and post trial care	#30	Provisions, if any, for ancillary and post-trial care, and for compensation to those who suffer harm from trial participation	N/A
Dissemination policy: trial results	#31a	Plans for investigators and sponsor to communicate trial results to participants, healthcare professionals, the public, and other relevant groups (eg, via publication, reporting in results databases, or other data sharing arrangements), including any publication restrictions	11
Dissemination policy: authorship	#31b	Authorship eligibility guidelines and any intended use of professional writers	N/A
Dissemination policy: reproducible research	#31c	Plans, if any, for granting public access to the full protocol, participant-level dataset, and statistical code	11
**Appendices**			
Informed consent materials	#32	Model consent form and other related documentation given to participants and authorised surrogates	N/A
Biological specimens	#33	Plans for collection, laboratory evaluation, and storage of biological specimens for genetic or molecular analysis in the current trial and for future use in ancillary studies, if applicable	N/A

For peer review only

The SPIRIT Explanation and Elaboration paper is distributed under the terms of the Creative Commons Attribution License CC-BY-NC. This checklist was completed on 20. April 2021 using https://www.goodreports.org/, a tool made by the EQUATOR Network in collaboration with Penelope.ai

For peer review only

BMJ Open

Evaluating the feasibility and acceptability of virtual group exercise for older adults delivered by trained volunteers: the ImPACt study protocol

Journal:	*BMJ Open*
Manuscript ID	bmjopen-2021-052631.R2
Article Type:	Protocol
Date Submitted by the Author:	22-Dec-2021
Complete List of Authors:	Lim, Stephen; University of Southampton, Academic Geriatric Medicine; University of Southampton, NIHR ARC Wessex<br>Meredith, Samantha; University of Southampton, Academic Geriatric Medicine; University of Southampton, NIHR ARC Wessex<br>Agnew, Samantha; The Brendoncare Foundation<br>Clift, Esther; Southern Health NHS Foundation Trust<br>Ibrahim, Kinda; University of Southampton, Academic Geriatric Medicine, Faculty of Medicine; University of Southampton, NIHR ARC Wessex<br>Roberts, Helen; University of Southampton, Academic Geriatric Medicine; University of Southampton, NIHR ARC Wessex
**Primary Subject Heading**:	Geriatric medicine
Secondary Subject Heading:	Qualitative research, Sports and exercise medicine
Keywords:	GERIATRIC MEDICINE, QUALITATIVE RESEARCH, REHABILITATION MEDICINE

SCHOLARONE™
Manuscripts

Title: Evaluating the feasibility and acceptability of virtual group exercise for older adults delivered by trained volunteers: the ImPACt study protocol

Protocol version 1.0

Corresponding author: Dr Stephen Eu Ruen Lim

Email: s.e.lim@soton.ac.uk

Address: Mailpoint 807, Academic Geriatric Medicine, University of Southampton,

Southampton General Hospital, Tremona Road,

Southampton, United Kingdom, SO16 6YD

Tel: 02381206131

Co-authors:

Dr Samantha Jane Meredith; University of Southampton, Southampton, UK

Samantha Agnew; The Brendoncare Foundation, Winchester, UK

Esther Clift; Southern Health NHS Foundation Trust, Southampton, UK

Dr Kinda Ibrahim; University of Southampton, Southampton, UK

Professor Helen C Roberts; University of Southampton, Southampton, UK

Key words:

Exercise, Older adults, volunteer, physical activity

Allowed word count: 4000

Word count: 3911 words

For peer review only

Abstract

Introduction

Physical activity is important for healthy ageing. Despite strong evidence on the benefits of physical activity for health and well-being, physical inactivity remains a significant problem among older adults. This study aims to determine the feasibility and acceptability of implementing an online volunteer-led group exercise for older adults.

Methods

A quasi-experimental mixed methods approach will be used in this study. A training programme will be developed to train volunteers to deliver online group exercises to older adults aged >65 years(n=30). The primary outcome is the feasibility of implementing the intervention. This will be assessed by the number of volunteers recruited, trained, and retained at the end of the study, and the number of exercise sessions delivered and completed by participants. Secondary outcomes include physical activity levels measured using the Community Health Model Activities Program for Seniors(CHAMPS) questionnaire, Barthel Index, EQ-5D-5L as a measure of health-related quality of life, SARC F to determine sarcopenia status, and PRIMSA-7 to determine frailty status. Outcomes will be measured at baseline and at 6 months.

Qualitative interviews will be conducted with volunteers(n=5), older adults(n=10), and family members(n=5) to explore their views on the intervention.

Analysis

Simple descriptive statistics will be used to describe participant characteristics, the feasibility of the study and the impact of the intervention on health outcomes. Parametric(t-test) or non-parametric(Mann-Whitney U test) statistics will be used to analyse continuous variables. Chi-square test will be used for categorical variables. Qualitative data will be analysed using an inductive thematic analysis approach.

Ethics and Dissemination

This study received ethical approval from the University of Southampton Faculty of Medicine Ethics Committee and Research Integrity and Governance committee (ID: 52967.A1). Study findings will be made available to service users, voluntary organisations and other researchers who may be interested in implementing the intervention.

For peer review only - http://bmjopen.bmj.com/site/about/guidelines.xhtml

Trial registration number

Clinicaltrials.gov: NCT04672200

For peer review only

For peer review only - http://bmjopen.bmj.com/site/about/guidelines.xhtml

Article summary

Strengths and limitations of this study

- This is a novel study exploring the use of trained volunteers to deliver online group exercises for older adults.
- It addresses the issue of physical inactivity among older people, a problem which has been exacerbated by the COVID-19 pandemic.
- A mixed methods approach will support the development of a toolkit for future implementation.
- For safety reasons, volunteers will deliver seated exercises in online groups.
- The exclusion of older adults who are not able to provide consent limits the generalisability of this study.

For peer review only

For peer review only - http://bmjopen.bmj.com/site/about/guidelines.xhtml

Introduction

Physical activity is important for healthy ageing. Some of the benefits of regular physical activity for older people include reduction in falls(1), better physical functioning(2), and improvement in well-being(3). The recommended physical activity level for older adults aged 65 years and above is to achieve 150 minutes of moderate physical activity in a week, with 2 days of strength training(4). This is often expressed as 30 minutes of brisk walking or equivalent activity five days a week, although 75 minutes of vigorous intensity activity spread across the week, or a combination of moderate and vigorous activity are also suggested(5).

Despite strong evidence for the benefits of physical activity, physical inactivity remains a significant problem among older adults worldwide(6). Studies in the UK have shown that less than 7% of community-dwelling older adults achieve the recommended weekly physical activity level(7, 8). The COVID-19 pandemic has exacerbated the problem of physical inactivity(9). Rules on social distancing and shielding of vulnerable groups have resulted in increased physical inactivity among older people, with potentially widespread deconditioning.

The rise of the use of technology during the pandemic may provide a solution to address the problem of physical inactivity among older people. Older adults are embracing modern technology more than ever before and are increasingly familiar with the use of devices and online platforms to stay in touch with their family members and friends(10, 11). This presents the opportunity to develop online physical activity interventions for older adults to promote increased physical activity. A review of reviews conducted by McGarrigle et al. found low to moderate evidence that physical activity interventions delivered through the internet and related technologies may be effective in increasing physical activity among older adults in the short term(12). Online group exercises have been shown to be feasible for older adults(13). However, there is little evidence on the use of trained volunteers to lead such exercise groups.

Volunteers play an integral part within many health and social care organisations. They enrich patient experience, help deliver high quality services, and support communities in living healthier lives(14). A report by the King's Fund on volunteering has highlighted that the support volunteers provide can be of particular value to those who rely most heavily on services, such as people with multiple long-term conditions or mental health problems(15). There is increasing evidence to suggest that with proper training, volunteers can take on more direct roles in supporting older adults particularly with nutrition(16) and mobility(17).

For peer review only - http://bmjopen.bmj.com/site/about/guidelines.xhtml

The Southampton Mobility Volunteer study (n=100, mean age 86 years) showed that volunteers can be trained to deliver chair-based exercises to older adults admitted to hospital(17). The intervention was shown to be safe, and acceptable to older adults and healthcare professionals, with evidence of improvement in daily step count among patients who received the intervention. The median age of the volunteers in the study was 32 years (interquartile ratio of 17 -62 years). A systematic review of 12 studies found some evidence suggesting that volunteer-led physical activity interventions that include resistance exercise training, can improve outcomes of community-dwelling older adults including functional status, frailty status and reduction in fear of falls(18). However, knowledge gaps still exist on how best to recruit, train, and retain volunteers to deliver physical activity interventions routinely. This study addresses this knowledge gap by exploring facilitators and barriers to the implementation of volunteer-led group exercises for older adults attending social clubs.

Older adults who volunteer to lead physical activity interventions also benefit from their volunteering, with one study showing evidence of improved physical activity levels(19). Older volunteers also benefit from the social interaction that volunteering brings, with reduced feelings of loneliness and having a sense of achievement(20). The use of trained volunteers to deliver online physical activity interventions remains an under-researched area and this study addresses the important knowledge gap on the feasibility and acceptability of such interventions(21).

Aims

This study aims to determine the feasibility and acceptability of training volunteers to deliver online group exercises to community-dwelling older adults. The specific objectives of this study are to:

1. Develop a training programme for volunteers to lead online group exercises
2. Determine the feasibility of recruiting, training and retaining volunteers to deliver the online intervention
3. Determine the acceptability of the online intervention to volunteers, older adults, and their carers
4. Explore barriers and facilitators to the implementation of the online intervention

For peer review only - http://bmjopen.bmj.com/site/about/guidelines.xhtml

Methods and Analysis

Study design

A quasi-experimental mixed methods approach will be used to determine the feasibility and acceptability of implementing online volunteer-led exercise sessions in community clubs. The study will be conducted at community-clubs focussed on promoting social interaction among older people. Feasibility studies are used to determine whether an intervention is appropriate for further evaluation, to determine sample sizes for controlled trials and to assess whether the ideas and findings can be shaped to be relevant and sustainable. In this study, the feasibility of training volunteers to promote increased physical activity among community-dwelling older adults will be assessed. The acceptability of the intervention will be examined through qualitative interviews to explore the views and experiences of volunteers, older adults participating in the study, and their carers or family members. The impact of the intervention on physical activity levels and functional outcomes will also be measured. This will help inform the sample size calculation for a future effectiveness trial.

Participant recruitment

Volunteer recruitment

The research team will be invited by club staff to attend the virtual clubs to introduce the study. Club volunteers and members who are interested in leading the group exercise will indicate their interest to staff members or approach the research team directly. To lead the group exercise, volunteers must be physically able to perform the exercises themselves and be able to provide written consent. This will be assessed at the training sessions by the trainer (SM).

The participant information sheet and the consent form will be sent through the post. Club members are invited to contact the research team for further discussions about the study. Once the research team has received the signed consent form, volunteers will be invited to participate in the online training. To deliver online group exercise to 30 exercise participants, it is estimated that at least 12 volunteers will be needed to lead 6 online groups. Volunteers will be paired up to lead and deliver the intervention.

Volunteer training

For peer review only - http://bmjopen.bmj.com/site/about/guidelines.xhtml

The volunteer training programme will be led by a member of the research team with a background in sport and exercise science. The training programme was developed based on clinical expertise from therapists and current evidence including experience from a recently completed hospital-based study(17).

The training will be delivered online through Zoom. Each volunteer will receive two group training sessions, lasting an hour each, and further one to one sessions will be offered to volunteers who wish to have further training. At the first training session, volunteers are given an overview on the importance of physical activity. They are introduced to their role in greater detail and given the opportunity to ask questions. They will be taught the exercise protocol, including the warm up and cool down exercises. The trainer will demonstrate the exercises and provide guidance on how to perform the exercises accurately. They will also be taught the purpose and benefits of each exercise performed. Volunteers will receive training on how to lead the group well, with practical guidance such as being confident, speaking with a clear voice, pacing themselves, and providing positive encouragement to the participants. This is then followed by a practical session where the volunteers will practice leading the exercise among the training group members. The training session will also include key aspects such as personal safety and safety of participants. Examples of personal safety include ensuring appropriate clothing and footwear, having good ventilation in the room, staying well-hydrated, and adopting a correct posture whilst performing the exercises. Volunteers will be trained to coach the participants, ensuring that the exercises are performed safely and effectively to prevent injuries. They will receive training on how to document adverse events that occur during the exercise classes. They will also be given guidance on how to set up their digital devices. Volunteers will receive a training booklet containing the exercises and safety instructions, as well as an exercise video to support their training. Competency assessments based on a checklist will be conducted by the research team to ensure that volunteers are competent in delivering the exercises online (supplement 1).

The emphasis of the second training session will be to provide opportunity for the volunteers to practice leading the group sessions. The trainer will observe the volunteers as they lead the exercises and provide feedback to ensure that they are performed accurately. They will also be given advise on how to set up the exercise space to ensure there is adequate space for the exercises to be performed safely, and that the device is optimally set up for participants to see them performing the exercises clearly.

Throughout the study period, fidelity checks will be conducted to ensure that the volunteers are delivering high quality exercise sessions. The fidelity checks will comprise assessment of the

For peer review only - http://bmjopen.bmj.com/site/about/guidelines.xhtml

volunteer exercise delivery including the safe and effective demonstration and instruction of the warm-up, strength exercise, and cool down components of the exercises. A member of the research team will attend the group online sessions as an observer to conduct the checks. Volunteers who require further support in delivering the exercises will be offered additional coaching by the research team to ensure that the exercises are delivered correctly.

Regular meetings for the volunteers will be held throughout the study period to provide them with ongoing support from the research team and the organisation running the online clubs. The group meetings will provide an avenue for volunteers to discuss and share their experiences, ask questions, and provide suggestions to improve the intervention.

Exercise participant recruitment

Club members who are interested in participating in the online group exercise will indicate their interest to club staff or approach the research team directly. Participant information sheet and consent forms will be posted to club members.

The inclusion criteria for the group exercise participants are: adults aged 65 years and older, able to walk with or without a walking aid, and able to provide written consent (Table 1). Participants who are actively participating in other online exercise classes will be excluded. The aim is to recruit 30 older adults attending social clubs who do not engage in regular exercise.

Intervention

The intervention consists of a once weekly volunteer-led online group home-based exercise, for a duration of 30 minutes. The online group will be led by volunteers and hosted on the digital platform Zoom(22). To comply with the social club's online insurance policy and to minimise the risk of injury to participants, the intervention will only include seated exercises. The seated exercises will focus on strengthening upper and lower limbs and enhancing whole body range of motion and flexibility, tailored specifically to meet the needs of older adults through considering appropriate FITT (frequency, intensity, time, type) principles and exercise adaptations (e.g., in response to joint pain and discomfort). Participants will be encouraged by the volunteers to progress by increasing repetitions and to gently improve range of motion. No supplementary equipment is needed for the online exercises. In addition to the group exercise sessions, participants will also be encouraged to increase mobility and physical activity levels through use of a home activity booklet, including a

For peer review only - http://bmjopen.bmj.com/site/about/guidelines.xhtml

physical activity diary. The exercise sessions will be piloted in one club before being extended to other clubs.

Outcomes measures

Volunteer characteristics such as age, gender, employment status, previous volunteering experience, and experience in delivering group exercises will be recorded. Participant characteristics including age, gender, domicile status, body mass index, co-morbidities, cognition (telephone mini-mental state examination) and number of medications will be recorded.

Primary outcomes

The primary outcome of this study is the feasibility of implementing volunteer-led online group exercise for older adults. This will be assessed by determining:

1. the number of volunteers recruited, trained, and retained at the end of the study,
2. the number of physical activity sessions delivered and completed by participants (adherence), and
3. the fidelity of the exercise intervention delivered by volunteers.

Secondary outcomes

The secondary outcome measures include measurement of physical activity levels and performance of activities of daily living. Physical activity levels will be measured using the Community Health Model Activities Program for Seniors (CHAMPS) questionnaire(23). The questionnaire assesses self-reported weekly frequency and duration of various physical activities typically undertaken by older adults, and is a validated measure of physical activity for older people(24). Performance of activities of daily living (ADL) will be measured using the Barthel Index(25). The Barthel Index measures ADL including transfers, walking, stairs, toilet use, dressing, feeding, bladder control, bowel control, grooming and bathing, to give a maximum total score of 100. It is a validated measure of ADL that is recommended for use in research among older people(26). The EQ-5D-5L measure developed by the EuroQol group will be used to determine health-related quality of life(27). The measure comprises five dimensions: mobility, self-care, usual activities, pain/discomfort, and anxiety/ depression. Each dimension is given a 1- digit score and the digits for the five dimensions are combined unto a 5-digit number that describes the patient's health state. The measure is commonly used in research among older people(28, 29). The SARC-F questionnaire will be used as a measure to assess for sarcopenia(30). According to recent guidelines from the European Working Group on

For peer review only - http://bmjopen.bmj.com/site/about/guidelines.xhtml

Sarcopenia in Older People, SARC-F is the recommended tool for sarcopenia case finding (31). It comprises five components: strength, assistance in walking, rise from a chair, climbing stairs, and falls. The scores range from 0 to 10, with a score of equal to or greater than four being predictive of sarcopenia and poor outcome. PRISMA-7 will be used to characterise participants' frailty status(32). The questionnaire contains seven items with a positive score of three or more indicating frailty. The PRISMA-7 tool has been shown to have high accuracy in identifying frail older adults in the community setting(33).

These outcome measures will be recorded at baseline, using remote assessment formats (e.g., telephone-based) and repeated at 6 months. Cost analysis will be conducted to determine the cost of implementing this intervention. The analysis will include the cost of training the volunteers, taking into consideration the time needed to deliver the group training sessions and the individual competency assessments, and the provision of training materials.

Safety and adverse events

To determine the safety of the intervention, adverse events that occur as a result of the intervention will be recorded. Any injuries or symptoms developed directly as a result of the exercises will be recorded as an adverse event. Volunteers will be trained to document any adverse events and report it back to the research team. The clubs also have well established procedures for responding to incidents and accidents including access to emergency contacts of participants.

Acceptability of the intervention

Interviews will be conducted with older adults, their family members or carers, volunteers, and those involved in recruiting participants and training volunteers, to determine the acceptability of the training and the intervention and to explore barriers and enablers to the implementation of the intervention. Interviews will be semi-structured to help guide conversation whilst allowing participants the flexibility to elaborate and reflect on any meaningful experiences(34). The interviews will be conducted by a member of the research team (SM) who is experienced in qualitative interviews. The interviews will be conducted within the first 2 months of the exercise groups to understand how the intervention is being delivered and whether any modifications to the intervention are needed. Further interviews will be conducted towards the end of the study period (at 6 months) to understand stakeholders' lived experience with the intervention and to explore barriers and facilitators for engagement and delivery of the intervention. This will ensure that participants' views are captured during the earlier stages of the intervention, and when the groups are well-established.

For peer review only - http://bmjopen.bmj.com/site/about/guidelines.xhtml

Older adults will be selected by purposive sampling to share their thoughts and views regarding the implementation of the volunteer-led physical activity session(35). To ensure that a wide range of views are included in the interviews, participants will be selected to include male and female participants, different clubs, and a representative age range. The aim is to interview at least two participants per club, with a total number of 10 participants. Family members and carers (n=5) of the exercise participants will also be invited to participate in the interviews. Their views and experience will provide valuable insight into the acceptability of the intervention and the potential barriers and enablers for a future wider implementation. All interviews will be conducted via telephone or online (e.g., Zoom, Skype) depending upon participant preference.

Semi-structured interviews will also be conducted with volunteers leading the intervention. Discussions among volunteers are likely to generate a wider perspective of the implementation process and may provide insight into the group narrative on their experiences in delivering the intervention and their interaction with the research participants(36, 37). The aim is to interview at least one volunteer from each club, with a total number of five volunteers.

The semi-structured interviews will consist of several key open-ended questions that help define the areas explored but allow the interviewer or interviewee to expand and diverge with the aim of pursuing or developing an idea with more depth. The interview schedules will be underpinned by Normalisation Process Theory (NPT)(38). The interviews will be audio-recorded for data collection purposes.

Normalisation Process Theory

The NPT provides a set of sociological tools to understand and explain the social processes through which new or modified practice of thinking, enacting, and organizing work are operationalized in healthcare and other institutional settings(38). The use of an implementation theory is useful as it helps researchers identify, describe and explain important elements of the implementation process(39). NPT was chosen as the framework that underpinned this study as it was systematic in its approach, easy to understand and provided a structured framework to evaluate the implementation process of this study. The online toolkit available for this implementation theory also made the application of NPT convenient and practical(40). The interview schedules were developed based on the four components of NPT:

I. Coherence: meaning and sense-making.
II. Cognitive participation: this explores the likelihood of all participants being committed and engaged with the intervention.

For peer review only - http://bmjopen.bmj.com/site/about/guidelines.xhtml

III. Collective action: the consideration of the work that participants do to make the intervention function.

IV. Reflexive monitoring: which involves participants appraising or reflecting on the intervention.

The questions will be framed to explore each component of the NPT in greater detail, with the aim to determine factors that facilitated or inhibited these four key processes which are essential for successful implementation and adoption of the intervention. The first section of the interview guide will focus on interviewees' views on the relevance and importance of physical activity in general, and in particular, the online exercises. Next, participants' views on factors that encouraged or prevented them from being committed and engaged with the online group exercise will be explored. Volunteers, staff members, older adults, and their carers' experience of the exercise group and how the stakeholders interacted with each other will then be explored to better understand the impact of the group dynamics on the intervention. Interviewees will then be encouraged to reflect on what worked well with the intervention and explore aspects within the intervention that could be improved or require modification.

Data analysis plan

Quantitative data analysis

Data collected will be double entered into a secured database for analysis. Statistical analysis will be conducted using the statistical software SPSS Statistics 25. Descriptive statistics -median (IQR); mean (SD); number (%) – will be used to analyse the numbers of volunteers recruited, trained, and retained, as well as the type and extent of progression of the seated exercise, and duration of their activity, and patients' adherence to the intervention. Analysis of the above outcome measures will be used to develop an assessment of the feasibility of delivering this intervention. Adherence to the intervention and reasons for non-adherence will be reported.

Outcome measures recorded at baseline will be compared to measurement at 6 months to determine if the PA intervention had an impact on physical activity levels measured by CHAMPS, functional outcomes including performance in activities of daily living (Barthel Index), quality of life (EQ-5D-5L l), frailty status (PRISMA 7) and sarcopenia status (SARC-F). The distribution of each outcome measure will be assessed for normality. Depending on the skew of the data, parametric (t-test) or non-parametric (Mann-Whitney U test) statistics will be used for continuous variables. Chi-square test will be used for categorical variables.

Qualitative data analysis

For peer review only - http://bmjopen.bmj.com/site/about/guidelines.xhtml

Data collected from the interviews will be transcribed verbatim and analysed with an inductive thematic analysis approach(41). This approach includes: Phase 1 – familiarising with the data, Phase 2 – generating initial codes, Phase 3 – searching for themes, Phase 4 – reviewing themes, Phase 5 – defining and naming themes, and Phase 6 – producing the report. Analysis of qualitative data will be conducted using NVivo. A sample of transcribed text will be read and coded separately and then together by two researchers to create a coding manual that applies to the entire set of transcripts. The codes will be analysed to generate concepts and ideas to determine the acceptability of the intervention, and to identify facilitators and barriers to the implementation process. The codes act as tags or labels to help catalogue key concepts embedded within the raw data. From the codes, themes will be developed to reflect the views and experiences of the community-dwelling older adults and volunteers regarding the online physical activity intervention.

A toolkit that will support a wider implementation of the intervention will be developed. It will be a written manual which contains the training programme, exercises, competency checklists, and information on how best to implement the intervention, taking into consideration the barriers and facilitators determined through the qualitative interviews.

Patient and public involvement

PPI involvement was sought in the development of this protocol. Visits were made to two clubs and discussions were held among club volunteers and club attendees about this project. Their views on what type of activities and the frequency of the activities were explored. Club attendees were also asked about what outcomes were important to them, which will be included in the study.

SA who is a co-applicant in this study, will also represent the views of club members and volunteers. She is the head of clubs for the organisation and is experienced in coordinating clubs. She has an overview on how the clubs are conducted and her input on the practical aspects of implementing the intervention will be invaluable. Her practical experience gained through interaction with club members and volunteers will help ensure that their views are represented.

Two additional PPI representatives will be invited to be part of the study management group led by the PPI lead for the Ageing and Dementia theme within the Wessex ARC. This group will aim to meet up quarterly to discuss the study progress and will be involved in the design of the research, discussion of the results and dissemination of research findings. PPI has also been involved in the preparation of public-facing materials and study documents.

Strengths and limitations of the study

For peer review only - http://bmjopen.bmj.com/site/about/guidelines.xhtml

This study explores a novel approach of training volunteers to deliver online group exercise to older adults. Application of a mixed methods approach in this study will help determine the feasibility of the intervention and explore barriers and facilitators to a successful implementation of the intervention. However, a limitation of the study is the lack of a control group. Recruitment of older adults will be through established social clubs, in which recruiting some older adults and excluding others from participating in the exercise groups would be a challenge. Additionally, as this is a feasibility study, we will use a range of social clubs of varying sizes, and client groups with different age ranges and physical abilities, to explore different approaches to the intervention rather than including a control group. Nevertheless, this study has been designed to assess the feasibility and acceptability of the intervention with a view to informing the development of a toolkit to support the wider implementation of the intervention and a future controlled experimental design. This study excluded older adults who are not able to walk. Patients who are bed-bound will not be able to participate in the seated exercises without additional support. This additional support cannot be met by this study. As the intervention is delivered remotely, participants must have a level of independence to participate safely. Additionally, one of the key outcome measure is physical activity levels and older adults who are bed-bound will have minimal activity levels.

Ethics and dissemination

Ethical and safety considerations

This study has received ethical approval from the University of Southampton Faculty of Medicine Ethics Committee and Research Integrity and Governance committee (ID: 52967.A1) Data will be stored on university repository PURE and will be made available to other researchers on request.

Dissemination plan

Findings from this study will be published in peer-reviewed journals and presented at national and international scientific platforms. A toolkit will be developed to support future implementation plans.

Author Contributions

SL, EC, SA, KI, and HCR were involved in the conception and trial design. SM will lead the volunteer training and be involved in data collection, and analysis. All authors contributed in the preparation of this article.

For peer review only - http://bmjopen.bmj.com/site/about/guidelines.xhtml

Funding statement

The National Institute for Health Research (NIHR) Applied Research Collaboration Wessex funded this research (Award number: not applicable). The views expressed are those of the authors and not necessarily those of the NHS, the NIHR or the Department of Health. SL, SM, KI, and HCR received support from the NIHR Applied Research Collaboration (ARC) Wessex and the University of Southampton. HCR received support from the NIHR Southampton Biomedical Research Centre. The University of Southampton NIHR Academic Clinical Lectureship scheme supported SL (Award number: CL2018-26-002).

Competing Interests

The authors have no competing interests to declare.

Table 1 Inclusion and exclusion criteria

Inclusion Criteria
Older adults aged > 65 years<br>Able to walk, with or without a walking aid<br>Able to provide written consent
**Exclusion Criteria**
Actively participating in other online exercise classes

For peer review only - http://bmjopen.bmj.com/site/about/guidelines.xhtml

References

1. Sherrington C, Fairhall N, Kwok W, Wallbank G, Tiedemann A, Michaleff ZA, et al. Evidence on physical activity and falls prevention for people aged 65+ years: systematic review to inform the WHO guidelines on physical activity and sedentary behaviour. International Journal of Behavioral Nutrition and Physical Activity. 2020;17(1):144.
2. Halaweh H, Willén C, Svantesson U. Association between physical activity and physical functioning in community-dwelling older adults. European Journal of Physiotherapy. 2017;19(1):40-7.
3. Fox KR, Stathi A, McKenna J, Davis MG. Physical activity and mental well-being in older people participating in the Better Ageing Project. European Journal of Applied Physiology. 2007;100(5):591-602.
4. Foster C, Reilly J, Jago R, Murphy M, Skelton D, Cooper A, et al. UK Chief Medical Officers' Physical Activity Guidelines. GOV.UK: UK Department of Health and Social Care 2019 07/09/2019.
5. Sparling PB, Howard BJ, Dunstan DW, Owen N. Recommendations for physical activity in older adults. BMJ. 2015;350(jan20 6):h100.
6. Cunningham C, R OS, Caserotti P, Tully MA. Consequences of physical inactivity in older adults: A systematic review of reviews and meta-analyses. Scandinavian journal of medicine & science in sports. 2020;30(5):816-27.
7. Harris TJ, Owen CG, Victor CR, Adams R, Cook DG. What factors are associated with physical activity in older people, assessed objectively by accelerometry? British journal of sports medicine. 2009;43(6):442-50.
8. Jefferis BJ, Sartini C, Lee IM, Choi M, Amuzu A, Gutierrez C, et al. Adherence to physical activity guidelines in older adults, using objectively measured physical activity in a population-based study. BMC public health. 2014;14(1):382-.
9. Wider impacts of COVID-19 on physical activity, deconditioning and falls in older adults. Public Health England. Available from: https://www.gov.uk/government/publications/covid-19-wider-impacts-on-people-aged-65-and-over. Last accessed: 05/10/2021
10. Marston HR, Genoe R, Freeman S, Kulczycki C, Musselwhite C. Older Adults' Perceptions of ICT: Main Findings from the Technology In Later Life (TILL) Study. Healthcare (Basel). 2019;7(3):86.
11. Barbosa Neves B, Franz R, Judges R, Beermann C, Baecker R. Can Digital Technology Enhance Social Connectedness Among Older Adults? A Feasibility Study. Journal of applied gerontology : the official journal of the Southern Gerontological Society. 2019;38(1):49-72.
12. McGarrigle L, Todd C. Promotion of Physical Activity in Older People Using mHealth and eHealth Technologies: Rapid Review of Reviews. J Med Internet Res. 2020;22(12):e22201.
13. Baez M, Khaghani Far I, Ibarra F, Ferron M, Didino D, Casati F. Effects of online group exercises for older adults on physical, psychological and social wellbeing: a randomized pilot trial. PeerJ. 2017;5:e3150.
14. Mundle C, Naylor C, Buck D. Volunteering in health and care in England: A summary of key literature. 2012. Available from: https://www.kingsfund.org.uk/sites/default/files/field/field_related_document/volunteering-in-health-literature-review-kingsfund-mar13.pdf. Last accessed: 05/10/2021
15. Naylor C, Mundle C, Weaks L, Buck D. Volunteering in health and care: Securing a sustainable future 2013 [28/07/2015]. Available from: http://www.kingsfund.org.uk/publications/volunteering-health-and-care. Last accessed: 05/10/2021
16. Roberts HC, De Wet S, Porter K, Rood G, Diaper N, Robison J, et al. The feasibility and acceptability of training volunteer mealtime assistants to help older acute hospital inpatients: the Southampton Mealtime Assistance Study. Journal of clinical nursing. 2014;23(21-22):3240-9.

For peer review only - http://bmjopen.bmj.com/site/about/guidelines.xhtml

17. Lim S, Ibrahim K, Dodds R, Purkis A, Baxter M, Rogers A, et al. Physical activity in hospitalised older people: the feasibility and acceptability of a volunteer-led mobility intervention in the SoMoVe study. Age and Ageing. 2020;49(2):283-91.
18. Lim SER, Cox NJ, Tan QY, Ibrahim K, Roberts HC. Volunteer-led physical activity interventions to improve health outcomes for community-dwelling older people: a systematic review. Aging Clin Exp Res. 2021;33(4):843-53.
19. Tan EJ, Xue Q-L, Li T, Carlson MC, Fried LP. Volunteering: a physical activity intervention for older adults--The Experience Corps program in Baltimore. J Urban Health. 2006;83(5):954-69.
20. Withall J, Thompson JL, Fox KR, Davis M, Gray S, Koning Jd, et al. Participant and Public Involvement in Refining a Peer-Volunteering Active Aging Intervention: Project ACE (Active, Connected, Engaged). Gerontologist. 2018;58(2):362-75.
21. Goehner A, Kricheldorff C, Bitzer EM. Trained volunteers to support chronically ill, multimorbid elderly between hospital and domesticity - a systematic review of one-on-one-intervention types, effects, and underlying training concepts. BMC Geriatr. 2019;19(1):126.
22. Zoom Available from: https://zoom.us/.
23. Hekler EB, Buman MP, Haskell WL, Conway TL, Cain KL, Sallis JF, et al. Reliability and validity of CHAMPS self-reported sedentary-to-vigorous intensity physical activity in older adults. J Phys Act Health. 2012;9(2):225-36.
24. Falck RS, McDonald SM, Beets MW, Brazendale K, Liu-Ambrose T. Measurement of physical activity in older adult interventions: a systematic review. British Journal of Sports Medicine. 2015.
25. Wade DT, Collin C. The Barthel ADL Index: A standard measure of physical disability? International Disability Studies. 1988;10(2):64-7.
26. Hopman-Rock M, van Hirtum H, de Vreede P, Freiberger E. Activities of daily living in older community-dwelling persons: a systematic review of psychometric properties of instruments. Aging Clinical and Experimental Research. 2019;31(7):917-25.
27. Rabin R, Charro Fd. EQ-SD: a measure of health status from the EuroQol Group. Annals of Medicine. 2001;33(5):337-43.
28. Pérez-Ros P, Vila-Candel R, Martin-Utrilla S, Martínez-Arnau FM. Health-Related Quality of Life in Community-Dwelling Older People with Cognitive Impairment: EQ-5D-3L Measurement Properties. Journal of Alzheimer's disease : JAD. 2020;77(4):1523-32.
29. Hulme C, Long AF, Kneafsey R, Reid G. Using the EQ-5D to assess health-related quality of life in older people. Age Ageing. 2004;33(5):504-7.
30. Ida S, Kaneko R, Murata K. SARC-F for Screening of Sarcopenia Among Older Adults: A Meta-analysis of Screening Test Accuracy. Journal of the American Medical Directors Association. 2018;19(8):685-9.
31. Cruz-Jentoft AJ, Bahat G, Bauer J, Boirie Y, Bruyere O, Cederholm T, et al. Sarcopenia: revised European consensus on definition and diagnosis. Age Ageing. 2019;48(1):16-31.
32. Raîche M, Hébert R, Dubois M-F. PRISMA-7: A case-finding tool to identify older adults with moderate to severe disabilities. Archives of Gerontology and Geriatrics. 2008;47(1):9-18.
33. Hoogendijk EO, van der Horst HE, Deeg DJH, Frijters DHM, Prins BAH, Jansen APD, et al. The identification of frail older adults in primary care: comparing the accuracy of five simple instruments. Age and Ageing. 2012;42(2):262-5.
34. Gill P, Stewart K, Treasure E, Chadwick B. Methods of data collection in qualitative research: interviews and focus groups. Br Dent J. 2008;204(6):291-5.
35. Palinkas LA, Horwitz SM, Green CA, Wisdom JP, Duan N, Hoagwood K. Purposeful sampling for qualitative data collection and analysis in mixed method implementation research. Administration and policy in mental health. 2015;42(5):533-44.
36. Guest G, Namey E, Taylor J, Eley N, McKenna K. Comparing focus groups and individual interviews: findings from a randomized study. International Journal of Social Research Methodology. 2017;20(6):693-708.

For peer review only - http://bmjopen.bmj.com/site/about/guidelines.xhtml

37. Bloor M, Frankland J, Thomas M, Robson K. Focus groups in social research. London: Sage Publications; 2001.
38. May CR, Mair F, Finch T, MacFarlane A, Dowrick C, Treweek S, et al. Development of a theory of implementation and integration: Normalization Process Theory. Implementation science : IS. 2009;4(1):29.
39. May C. Towards a general theory of implementation. Implementation Science. 2013;8(1):18.
40. Normalization Process Theory Toolkit [Available from: http://www.normalizationprocess.org/npt-toolkit/. Last accessed: 05/10/2021]
41. Braun V, Clarke V. Using thematic analysis in psychology. Qualitative Research in Psychology. 2006;3:77-101.

For peer review only

For peer review only - http://bmjopen.bmj.com/site/about/guidelines.xhtml

ImPACt Volunteer Competencies

These competencies have been developed to enhance club members care during online exercise sessions. They have been broken down into separate competencies to ensure you feel equipped to work with older adults to promote their activity, whilst maintaining the individuals and your own safety. Each box should be dated and signed by yourself and the supporting therapist or researcher.

Name: **Date started:** **Date completed:**

Skill / Knowledge	Criteria	Training given	Skill practiced with supervision on peers	Skill practiced with supervision on patient	Skill completed (signed by therapist / researcher)
Personal safety awareness	Wear appropriate clothing and footwear to be comfortable when exercising.				
	Ensure your exercise space is set up safely, including a sturdy chair, and the space is free of obstacles.				
	Ensure you are well enough to exercise and know when exercise should be avoided.				
Basic patient safety	Demonstrate what to do if a participant feels unwell. Stop exercise. Participants should not exercise if they have a fever or any new or worsening symptoms.				
	If participants have symptoms indicative of an emergency situation e.g., chest pain, dizziness, or shortness of breath that does not go away upon cessation of exercise, then call 999.				
	CALL FOR HELP IF YOU FEEL IT IS AN EMERGENCY<br>If there is an emergency situation alert a staff member, who will support you.				

For peer review only

	Staff members will have access to participants emergency contact info.				
	Demonstrate what to do if a participant experiences pain during exercise. Stop and review the participants position and technique. Offer an alternative exercise if pain persists, or suggest they miss out the exercise and rest. Advise they seek advice from their GP if pain persists.				
Pre exercise checks	Demonstrate proficiency with online set up and use of an online platform (e.g., Zoom).				
	Introduction to participants / group. Explain role and overview of exercise session.				
	Determine participants readiness and safety to exercise. How are you? How are you feeling?				
	Ensure participants have a safe exercise set-up, including a sturdy chair, water nearby for hydration, and the room is clear of obstacles.				
	Ensure patient has appropriate clothing and footwear to exercise.				
Prescribed exercises	To demonstrate an understanding of the main components of an exercise session, including warm up, conditioning, and cool down.				

For peer review only

	To prompt and demonstrate correct technique for lower limb chair exercises.				
	To prompt and demonstrate correct technique for upper limb chair exercises.				
	To demonstrate verbal coaching proficiency, including clear feedback on exercise technique and rationale to participants.				
	To demonstrate ability to encourage and motivate participants to exercise.				
	To demonstrate understanding of exercise progressions.				
Documentation	Proficiency completing participant registers.				
	Proficiency completing adverse events log.				

For peer review only

Reporting checklist for protocol of a clinical trial.

Based on the SPIRIT guidelines.

Instructions to authors

Complete this checklist by entering the page numbers from your manuscript where readers will find each of the items listed below.

Your article may not currently address all the items on the checklist. Please modify your text to include the missing information. If you are certain that an item does not apply, please write "n/a" and provide a short explanation.

Upload your completed checklist as an extra file when you submit to a journal.

In your methods section, say that you used the SPIRITreporting guidelines, and cite them as:

Chan A-W, Tetzlaff JM, Gøtzsche PC, Altman DG, Mann H, Berlin J, Dickersin K, Hróbjartsson A, Schulz KF, Parulekar WR, Krleža-Jerić K, Laupacis A, Moher D. SPIRIT 2013 Explanation and Elaboration: Guidance for protocols of clinical trials. BMJ. 2013;346:e7586

For peer review only

		Reporting Item	Page Number
**Administrative information**			
Title	#1	Descriptive title identifying the study design, population, interventions, and, if applicable, trial acronym	1
Trial registration	#2a	Trial identifier and registry name. If not yet registered,	2

		name of intended registry	
Trial registration: data set	#2b	All items from the World Health Organization Trial Registration Data Set	2
Protocol version	#3	Date and version identifier	1
Funding	#4	Sources and types of financial, material, and other support	14
Roles and responsibilities: contributorship	#5a	Names, affiliations, and roles of protocol contributors	1, 14
Roles and responsibilities: sponsor contact information	#5b	Name and contact information for the trial sponsor	1
Roles and responsibilities: sponsor and funder	#5c	Role of study sponsor and funders, if any, in study design; collection, management, analysis, and interpretation of data; writing of the report; and the decision to submit the report for publication, including whether they will have ultimate authority over any of these activities	14
Roles and responsibilities: committees	#5d	Composition, roles, and responsibilities of the coordinating centre, steering committee, endpoint adjudication committee, data management team, and other individuals or groups overseeing the trial, if applicable (see Item 21a for data monitoring committee)	N/A

Introduction

For peer review only

Background and rationale	#6a	Description of research question and justification for undertaking the trial, including summary of relevant studies (published and unpublished) examining benefits and harms for each intervention	4
Background and rationale: choice of comparators	#6b	Explanation for choice of comparators	N/A
Objectives	#7	Specific objectives or hypotheses	5
Trial design	#8	Description of trial design including type of trial (eg, parallel group, crossover, factorial, single group), allocation ratio, and framework (eg, superiority, equivalence, non-inferiority, exploratory)	6
**Methods: Participants, interventions, and outcomes**			
Study setting	#9	Description of study settings (eg, community clinic, academic hospital) and list of countries where data will be collected. Reference to where list of study sites can be obtained	6
Eligibility criteria	#10	Inclusion and exclusion criteria for participants. If applicable, eligibility criteria for study centres and individuals who will perform the interventions (eg,	8

For peer review only

		surgeons, psychotherapists)	
Interventions: description	#11a	Interventions for each group with sufficient detail to allow replication, including how and when they will be administered	8
Interventions: modifications	#11b	Criteria for discontinuing or modifying allocated interventions for a given trial participant (eg, drug dose change in response to harms, participant request, or improving / worsening disease)	8
Interventions: adherance	#11c	Strategies to improve adherence to intervention protocols, and any procedures for monitoring adherence (eg, drug tablet return; laboratory tests)	8
Interventions: concomitant care	#11d	Relevant concomitant care and interventions that are permitted or prohibited during the trial	N/A
Outcomes	#12	Primary, secondary, and other outcomes, including the specific measurement variable (eg, systolic blood pressure), analysis metric (eg, change from baseline, final value, time to event), method of aggregation (eg, median, proportion), and time point for each outcome. Explanation of the clinical relevance of chosen efficacy and harm outcomes is strongly recommended	9
Participant timeline	#13	Time schedule of enrolment, interventions (including any run-ins and washouts), assessments, and visits for participants. A schematic diagram is highly recommended (see Figure)	8

For peer review only

Sample size	#14	Estimated number of participants needed to achieve study objectives and how it was determined, including clinical and statistical assumptions supporting any sample size calculations	8
Recruitment	#15	Strategies for achieving adequate participant enrolment to reach target sample size	6, 8
**Methods: Assignment of interventions (for controlled trials)**			
Allocation: sequence generation	#16a	Method of generating the allocation sequence (eg, computer-generated random numbers), and list of any factors for stratification. To reduce predictability of a random sequence, details of any planned restriction (eg, blocking) should be provided in a separate document that is unavailable to those who enrol participants or assign interventions	N/A
Allocation concealment mechanism	#16b	Mechanism of implementing the allocation sequence (eg, central telephone; sequentially numbered, opaque, sealed envelopes), describing any steps to conceal the sequence until interventions are assigned	N/A
Allocation: implementation	#16c	Who will generate the allocation sequence, who will enrol participants, and who will assign participants to interventions	N/A

For peer review only

Blinding (masking)	#17a	Who will be blinded after assignment to interventions (eg, trial participants, care providers, outcome assessors, data analysts), and how	N/A
Blinding (masking): emergency unblinding	#17b	If blinded, circumstances under which unblinding is permissible, and procedure for revealing a participant's allocated intervention during the trial	N/A
**Methods: Data collection, management, and analysis**			
Data collection plan	#18a	Plans for assessment and collection of outcome, baseline, and other trial data, including any related processes to promote data quality (eg, duplicate measurements, training of assessors) and a description of study instruments (eg, questionnaires, laboratory tests) along with their reliability and validity, if known. Reference to where data collection forms can be found, if not in the protocol	8
Data collection plan: retention	#18b	Plans to promote participant retention and complete follow-up, including list of any outcome data to be collected for participants who discontinue or deviate from intervention protocols	9
Data management	#19	Plans for data entry, coding, security, and storage, including any related processes to promote data quality (eg, double data entry; range checks for data values).	12

For peer review only

		Reference to where details of data management procedures can be found, if not in the protocol	
Statistics: outcomes	#20a	Statistical methods for analysing primary and secondary outcomes. Reference to where other details of the statistical analysis plan can be found, if not in the protocol	12
Statistics: additional analyses	#20b	Methods for any additional analyses (eg, subgroup and adjusted analyses)	12
Statistics: analysis population and missing data	#20c	Definition of analysis population relating to protocol non-adherence (eg, as randomised analysis), and any statistical methods to handle missing data (eg, multiple imputation)	N/A
**Methods: Monitoring**			
Data monitoring: formal committee	#21a	Composition of data monitoring committee (DMC); summary of its role and reporting structure; statement of whether it is independent from the sponsor and competing interests; and reference to where further details about its charter can be found, if not in the protocol. Alternatively, an explanation of why a DMC is not needed	N/A
Data monitoring: interim analysis	#21b	Description of any interim analyses and stopping guidelines, including who will have access to these interim results and make the final decision to terminate the trial	N/A
Harms	#22	Plans for collecting, assessing, reporting, and managing solicited and spontaneously reported adverse events and other unintended effects of trial interventions or trial	10

For peer review only

		conduct	
Auditing	#23	Frequency and procedures for auditing trial conduct, if any, and whether the process will be independent from investigators and the sponsor	N/A
**Ethics and dissemination**			
Research ethics approval	#24	Plans for seeking research ethics committee / institutional review board (REC / IRB) approval	14
Protocol amendments	#25	Plans for communicating important protocol modifications (eg, changes to eligibility criteria, outcomes, analyses) to relevant parties (eg, investigators, REC / IRBs, trial participants, trial registries, journals, regulators)	N/A
Consent or assent	#26a	Who will obtain informed consent or assent from potential trial participants or authorised surrogates, and how (see Item 32)	6
Consent or assent: ancillary studies	#26b	Additional consent provisions for collection and use of participant data and biological specimens in ancillary studies, if applicable	N/A
Confidentiality	#27	How personal information about potential and enrolled participants will be collected, shared, and maintained in order to protect confidentiality before, during, and after the trial	6
Declaration of	#28	Financial and other competing interests for principal	15

For peer review only

interests		investigators for the overall trial and each study site	
Data access	#29	Statement of who will have access to the final trial dataset, and disclosure of contractual agreements that limit such access for investigators	14
Ancillary and post trial care	#30	Provisions, if any, for ancillary and post-trial care, and for compensation to those who suffer harm from trial participation	N/A
Dissemination policy: trial results	#31a	Plans for investigators and sponsor to communicate trial results to participants, healthcare professionals, the public, and other relevant groups (eg, via publication, reporting in results databases, or other data sharing arrangements), including any publication restrictions	14
Dissemination policy: authorship	#31b	Authorship eligibility guidelines and any intended use of professional writers	N/A
Dissemination policy: reproducible research	#31c	Plans, if any, for granting public access to the full protocol, participant-level dataset, and statistical code	14
**Appendices**			
Informed consent materials	#32	Model consent form and other related documentation given to participants and authorised surrogates	N/A
Biological specimens	#33	Plans for collection, laboratory evaluation, and storage of biological specimens for genetic or molecular analysis in the current trial and for future use in ancillary studies, if applicable	N/A

For peer review only

The SPIRIT Explanation and Elaboration paper is distributed under the terms of the Creative Commons Attribution License CC-BY-NC. This checklist was completed on 20. April 2021 using https://www.goodreports.org/, a tool made by the EQUATOR Network in collaboration with Penelope.ai

For peer review only
